# Modulation Of Microtubule Acetylation By The Interplay Of TPPP/p25, SIRT2 And New Anticancer Agents With Anti-SIRT2 Potency

**DOI:** 10.1038/s41598-017-17381-3

**Published:** 2017-12-06

**Authors:** Adél Szabó, Judit Oláh, Sándor Szunyogh, Attila Lehotzky, Tibor Szénási, Marianna Csaplár, Matthias Schiedel, Péter Lőw, Manfred Jung, Judit Ovádi

**Affiliations:** 1 Institute of Enzymology, Research Centre for Natural Sciences, Hungarian Academy of Sciences, Budapest, H-1117 Hungary; 2 Institute of Pharmaceutical Sciences, University of Freiburg, 79104 Freiburg im Breisgau, Germany; 3 Department of Chemistry, Chemistry Research Laboratory, University of Oxford, OX1 3TA Oxford, United Kingdom; 4 Department of Anatomy, Cell and Developmental Biology, Eötvös Loránd University, Budapest, H-1117 Hungary

## Abstract

The microtubule network exerts multifarious functions controlled by its decoration with various proteins and post-translational modifications. The disordered microtubule associated Tubulin Polymerization Promoting Protein (TPPP/p25) and the NAD^+^-dependent tubulin deacetylase sirtuin-2 (SIRT2) play key roles in oligodendrocyte differentiation by acting as dominant factors in the organization of myelin proteome. Herein, we show that SIRT2 impedes the TPPP/p25-promoted microtubule assembly independently of NAD^+^; however, the TPPP/p25-assembled tubulin ultrastructures were resistant against SIRT2 activity. TPPP/p25 counteracts the SIRT2-derived tubulin deacetylation producing enhanced microtubule acetylation. The inhibition of the SIRT2 deacetylase activity by TPPP/p25 is evolved by the assembly of these tubulin binding proteins into a ternary complex, the concentration-dependent formation of which was quantified by experimental-based mathematical modelling. Co-localization of the SIRT2-TPPP/p25 complex on the microtubule network was visualized in HeLa cells by immunofluorescence microscopy using Bimolecular Fluorescence Complementation. We also revealed that a new potent SIRT2 inhibitor (MZ242) and its proteolysis targeting chimera (SH1) acting together with TPPP/p25 provoke microtubule hyperacetylation, which is coupled with process elongation only in the case of the degrader SH1. Both the structural and the functional effects manifesting themselves by this deacetylase proteome could lead to the fine-tuning of the regulation of microtubule dynamics and stability.

## Introduction

Microtubules (MTs) have important functions in the cell, ranging from cell morphology maintenance to subcellular transport, cellular signalling, cell migration, and cell polarity^1–3^. The dynamic balance between actin contraction and MT extension regulates mammalian cell shape, division, and motility^4–6^. MTs are crucial for various complex biological processes, such as viral entry, inflammation, immunity, learning and memory in mammals^3^. Due to its indispensable functions, the MT network is an attractive and successful target for anticancer drugs. A number of anti-MT drugs have been reported to reduce tumour growth by depolymerization (e.g. vinca alkaloids) or hyperstabilization (e.g. taxanes) of the MT network^4–6^. Regarding cancer therapeutic aspects, the application of these drugs can cause dose-limiting toxicities, since they do not target cancer cells specifically. Up to now, several data have also been reported that post-translational modifications of tubulin and Microtubule Associated Proteins (MAPs) could be more specific targets by avoiding the disruption of all MT assemblies^4^.

Tubulin Polymerization Promoting Protein (TPPP/p25), a recently discovered MAP^7–9^, is a disordered protein with extended unstructured terminal segments straddling a flexible region, which includes important binding motives such as a zinc finger and a GTP consensus sequence^10,11^. A number of *in vitro* and cellular studies with wild type and recombinant mutants as well as their fluorescently labelled variants have revealed that the structural changes of the disordered TPPP/p25 are mediated by its dimerization and heterologous interactions with the bivalent zinc cation, GTP, mitogen-activated protein kinase 1 and histone deacetylase 6 (HDAC6), which significantly affect its tubulin polymerization promoting potency^10–16^. This feature of TPPP/p25 is tightly coupled with its physiological functions, namely, the modulation and coordination of the dynamics and stability of the MT network^7,9,17^. Under physiological conditions, TPPP/p25 is predominantly expressed in differentiated oligodendrocytes (OLGs)^18–20^, the major constituents of the myelin sheath. However, under pathological conditions it is enriched and co-localizes with α-synuclein in neuronal and oligodendroglial inclusions, which are characteristic of synucleinopathies; therefore TPPP/p25 has been proposed as a hallmark protein of synucleinopathies^21,22^.

The tubulin subunits are subjected to a number of post-translational modifications, such as acetylation, phosphorylation, tyrosination, polyglycylation and polyglutamylation^3,23^. The acetylation level of tubulin/MTs is controlled by opposing enzymatic activities of tubulin deacetylases (HDAC6 and SIRT2)^24,25^ and acetyltransferases^26,27^. The silent information regulator (SIRT) proteins belong to the Class III HDACs displaying nicotinamide adenine dinucleotide (NAD^+^)-dependent deacetylase activity. HDAC6 and SIRT2 both deacetylate the Lys-40 aa of α-tubulin, *in vitro* and *in vivo* ^24,25^. The inhibition of either HDAC6 or SIRT2 by siRNA or specific inhibitors such as Trichostatin A (TSA) and AGK2, respectively, induces hyperacetylation of the MT network suggesting that both enzymes function independently in tubulin deacetylation^25^. However, HDAC6 inhibition led to general MT hyperacetylation, whereas hyperacetylation induced by SIRT2 inactivation was proposed to be limited to perinuclear MTs, indicating that the two deacetylases might recognise specific structural contexts^28^.

The binding of TPPP/p25 to the tubulin deacetylases has been demonstrated^16,29^. We have reported more detailed studies with HDAC6 revealing that its inhibition by TSA results in hyperacetylation of the MT network but does not cause MT stabilization. In contrast to this, TPPP/p25, in addition to its bundling activity, causes extensive acetylation of α-tubulin at Lys-40 along the MT network and the level of tubulin acetylation increases by the expression of TPPP/p25 in HeLa, CHO10 and CG-4 cells^16^. Down-regulation of TPPP/p25 by specific siRNA results in a simultaneous decrease of TPPP/p25 and acetylated tubulin levels^16^. We have also shown that the TPPP/p25-induced HDAC6 inhibition influences the growth velocity of the MT plus ends as well as the cell motility^16^.

The human SIRT2 plays a major role in mitosis, during which its abundance increases dramatically^30^. 26 S proteasome inhibitors were reported to enhance SIRT2 level, indicating that it is degraded by the proteasome machinery. SIRT2 is a component of the myelin proteome^31,32^ similarly as TPPP/p25^18–20^. The endogenous SIRT2 level is relatively high in OLGs^33,34^; the enzyme is also abundant in neurons, where it functions as the predominant MT deacetylase^35^. Abnormal level of MT acetylation is linked mainly to cancer, but it was found in neurological and heart diseases as well, yielding important therapeutic implications^3^. Therefore, the mechanism that regulates SIRT2 expression and activity needs to be clarified at molecular and cellular levels in order to validate SIRT2 as a specific drug target.

The sirtuin rearranging ligands (SirReals) are a new class of highly selective and drug-like inhibitors of SIRT2^36^. High-resolution SIRT2-SirReal co-crystal structures have revealed their unique mechanism of inhibition, which is based on a ligand-induced structural rearrangement of the active site and the formation of a yet-unexploited binding pocket^36^. Application of potent SirReals led to tubulin hyperacetylation in HeLa cells and induced the destabilization of the checkpoint protein BubR1, consistent with *in vivo* SIRT2 inhibition^36–38^. Most recently, a SirReal-derived proteolysis targeting chimera (PROTAC) was developed, which was shown to be able to selectively initiate the proteasomal degradation of SIRT2^39^. Chemical structures of the SirReal inhibitors and the SirReal-derived PROTAC can be found in Supplementary Fig. S1. In comparison with their parental direct enzyme inhibitors, PROTACs can be very useful tools to distinguish between the effects that are linked to the catalytic activity of an enzyme and those that are associated with non-enzymatic protein-protein interactions.

In this paper we established the regulatory potency of TPPP/p25 on the SIRT2-derived deacetylation of the tubulin/MT network as well as on the inhibitory potency of chemical SIRT2 inhibitors/degrader leading to the hyperacetylation of the MT network.

## Results

### Assembly of SIRT2 with TPPP/p25 and/or tubulin

**Figure 1:**
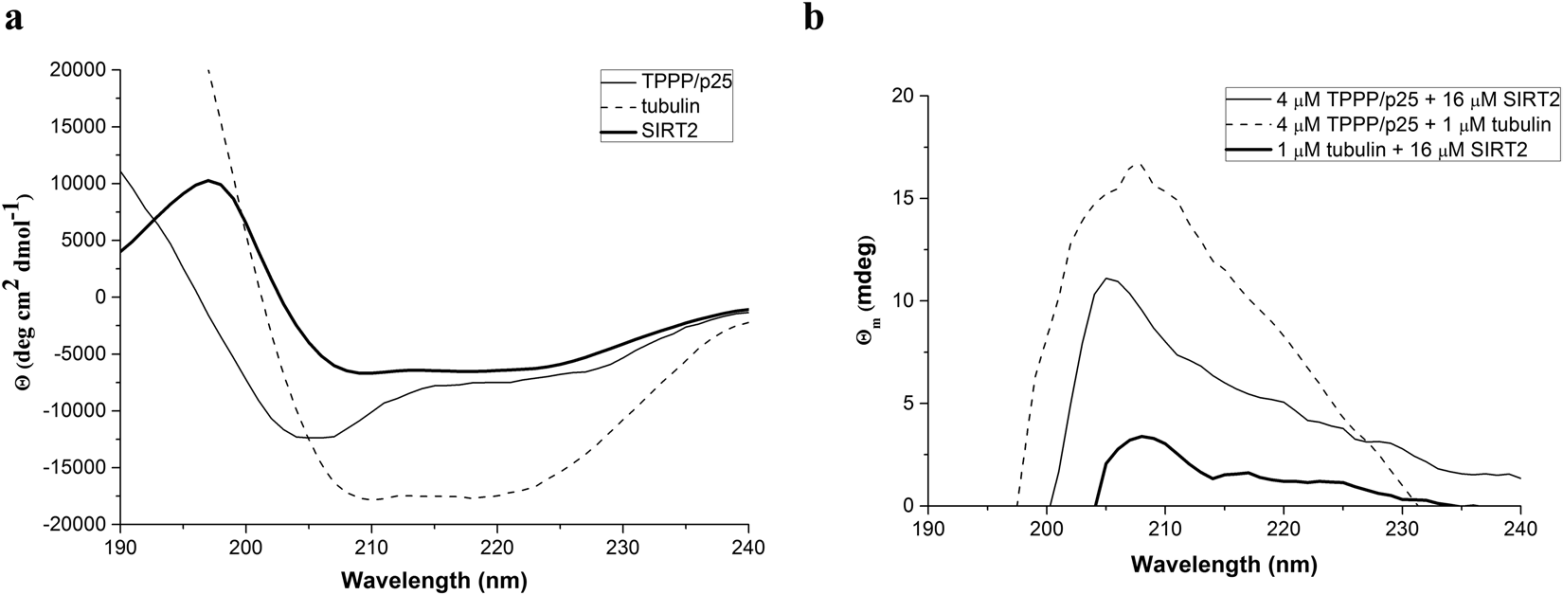
Secondary structural characteristics of SIRT2 and its complexes with tubulin and TPPP/p25 as detected by far-UV CD spectroscopy. (**a**) Normalized CD spectrum of SIRT2 (bold line), TPPP/p25 (solid line) and tubulin (dashed line) (n = 3); (**b**) Difference spectrum of SIRT2-tubulin (bold line), SIRT2-TPPP/p25 (solid line) and tubulin-TPPP/p25 (dashed line) (n = 3). Difference ellipticities were calculated by subtracting the ellipticities of the individual protein from that of their complexes. Concentrations of the proteins: 16 μM SIRT2, 4 μM TPPP/p25 and 1 μM tubulin.

The interaction of SIRT2 with TPPP/p25 as well as their associations to tubulin was characterized by circular dichroism (CD) and enzyme-linked immunosorbent assay (ELISA). As illustrated in Fig. 1a, the spectrum of the human recombinant SIRT2, similarly to that of tubulin, shows characteristic features typical for α-helical proteins with two prominent minima at 208 and 222 nm^40^; while the spectrum of TPPP/p25 corresponding to that of disordered proteins displays a minimum at 205 nm^7^. The pair-wise association of TPPP/p25 and SIRT2 generated a difference ellipticity spectrum with a maximum at nearly 207 nm indicating secondary structural changes likely in the disordered TPPP/p25 (Fig. 1b), similarly as reported for the complex of TPPP/p25 with tubulin^7^.

**Figure 2:**
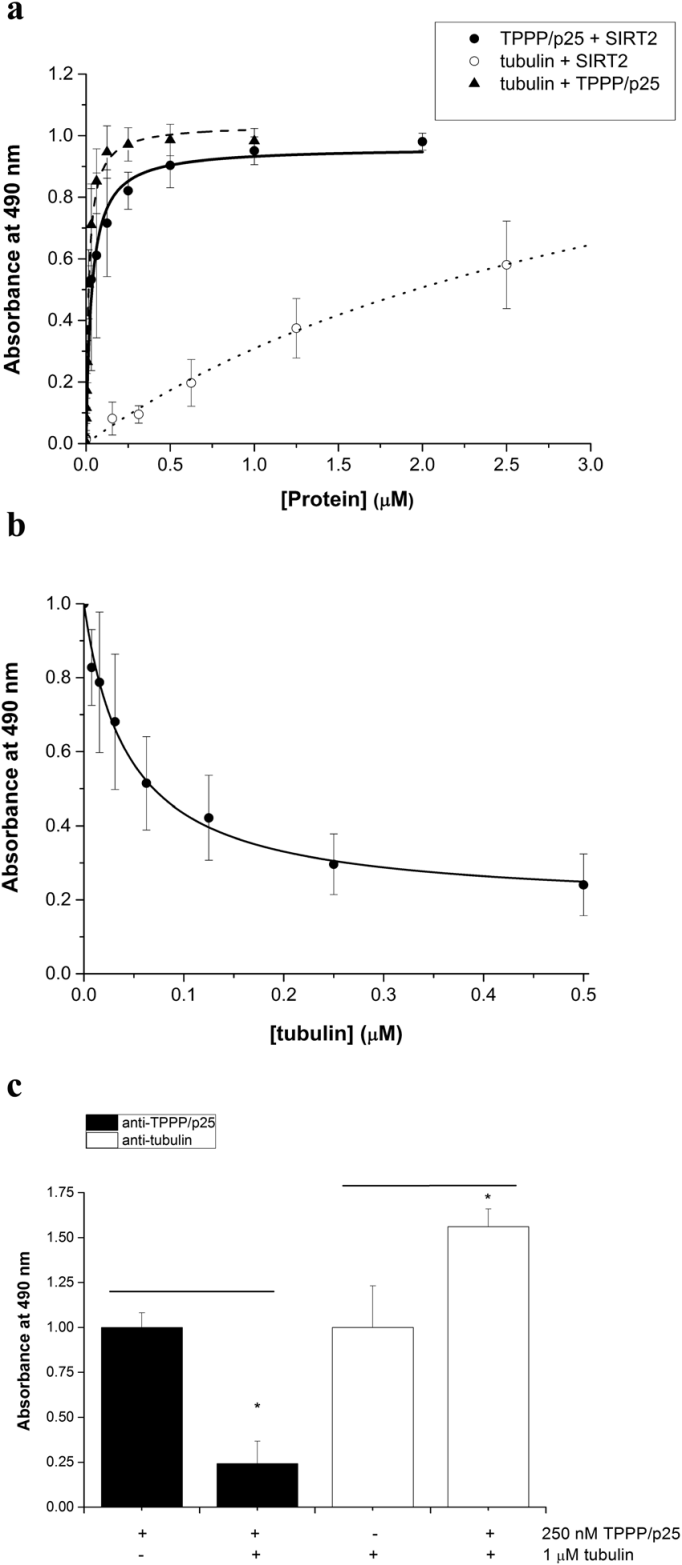
Quantification of the pair-wise interactions of SIRT2, TPPP/p25 and tubulin by ELISA. (**a**) Saturation curves of TPPP/p25 (●) and tubulin (○), when the plate was coated with SIRT2; and that of tubulin (▲), when the plate was coated with TPPP/p25. Concentrations of the coated proteins were 5 μg/ml. The data are presented as mean ± SD, n = 3 for TPPP/p25-SIRT2, n = 4 for tubulin-SIRT2, and n = 11 for TPPP/p25-tubulin. (**b**) Displacement of 125 nM TPPP/p25 from the coated SIRT2 by tubulin added at various concentrations detected by TPPP/p25 antibody. The data are presented as mean ± SD, n = 3. (**c**) Binding of TPPP/p25 (250 nM) and tubulin (1 μM) to the immobilized SIRT2 as detected by TPPP/p25 and tubulin antibodies, respectively. The data are presented as mean ± SD, n = 4. *p = 2.61E-04 when TPPP/p25 with tubulin compared to TPPP/p25 alone, and *p = 1.76E-02 when tubulin with TPPP/p25 compared to tubulin alone (two-sided, unpaired Student’s t-test).

The pair-wise interactions were quantified by ELISA (Fig. 2a): SIRT2 was immobilized on the plate, then TPPP/p25 or tubulin was added at various concentrations and their binding to SIRT2 was detected by specific TPPP/p25 or tubulin antibodies as described in the Materials and Methods. The apparent dissociation constant was K_d_ = 32.4 ± 4.6 nM for the association of TPPP/p25 and SIRT2, while it was significantly higher for SIRT2-tubulin (K_d_ ≥ 2 μM). These results, according to the earlier data^29^, indicate the preferential association of SIRT2 to TPPP/p25 as compared to tubulin.

These data were used to reveal the nature of the association of SIRT2 with tubulin in the presence of TPPP/p25. In the competitive ELISA experiments, SIRT2 was immobilized on the plate, then tubulin was added at various concentrations preincubated without or with TPPP/p25 of constant concentration. The SIRT2-bound TPPP/p25, as quantified by the immunopositivity, decreased by the increase of tubulin concentration suggesting a reduced binding potency of tubulin-bound TPPP/p25 to the immobilized SIRT2 (Fig. 2b). In another ELISA experiment, 1 μM tubulin or 0.25 μM TPPP/p25 or their preincubated mixture was added to the immobilized SIRT2, then both the bound tubulin and TPPP/p25 were detected by specific antibodies. Surprisingly, the comparison of these data with those obtained with binary complexes revealed that the presence of TPPP/p25 increased the amount of the bound tubulin, while the addition of tubulin reduced the binding of TPPP/p25 to SIRT2 (Fig. 2c). These findings show that i) both TPPP/p25 and SIRT2 are associated to tubulin; ii) the ratio of the binary and ternary complexes of SIRT2 depends on the concentration of the partners; iii) an excess tubulin level could produce an apparent competitive binding.

**Figure 3:**
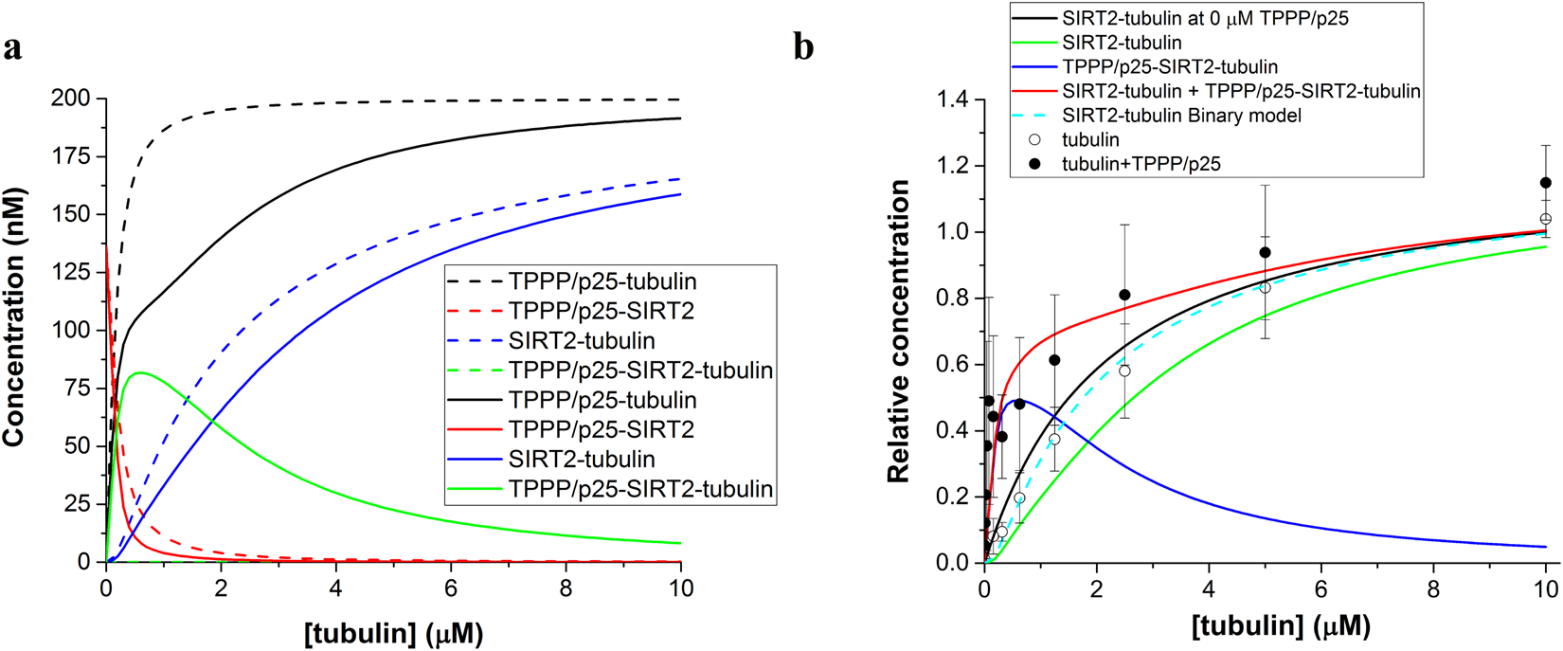
Mathematical model of the binary and ternary complexes and comparison with the ELISA experiment. (**a**) Concentration of the different complexes according to the binary (dashed lines) and ternary (solid lines) models. (**b**) SIRT2 was immobilized on the plate, then tubulin without (○) or with 200 nM TPPP/p25 (●) was added and the bound tubulin was detected by tubulin antibody. The data are presented as mean ± SD, n = 4 for tubulin, n = 3 for tubulin with TPPP/p25. SIRT2-tubulin at 0 μM TPPP/p25: relative concentration of SIRT2-tubulin complex without added TPPP/p25. Relative concentrations of SIRT2-tubulin, TPPP/p25-SIRT2-tubulin and the sum of SIRT2-tubulin and TPPP/p25-SIRT2-tubulin according to the ternary model, respectively, are shown. Relative concentration of SIRT2-tubulin according to the binary model is also displayed.

An experiment-based mathematical model was established to study the nature of the heteroassociations in relation to the formation of binary and ternary complexes depending on the tubulin concentration. Two types of models were evaluated: a competitive model assuming exclusively binary complexes, and a ternary complex model allowing the formation of the TPPP/p25-SIRT2-tubulin complex. The models include the dissociation constants of the binary complexes of tubulin, SIRT2 and TPPP/p25 determined experimentally as well as the total concentrations of the individual proteins (for details see Supplementary data and Supplementary Table S1). Figure 3 shows the binding of tubulin to the immobilized SIRT2 in the absence and presence of TPPP/p25 (cf. Fig. 2c) as well as the theoretical curves. The comparison of the computed and the experimental data of the ELISA experiments referring to the concentration-dependent binding of tubulin to SIRT2 in the presence of TPPP/p25 underlines the necessity of ternary complex formation to obtain appropriate fitting. The quantitative data determining the nature of the complexes render the evaluation of the ratio of the binary/ternary complexes possible.

### SIRT2 reduces the TPPP/p25-promoted tubulin assembly *in vitro*

**Figure 4:**
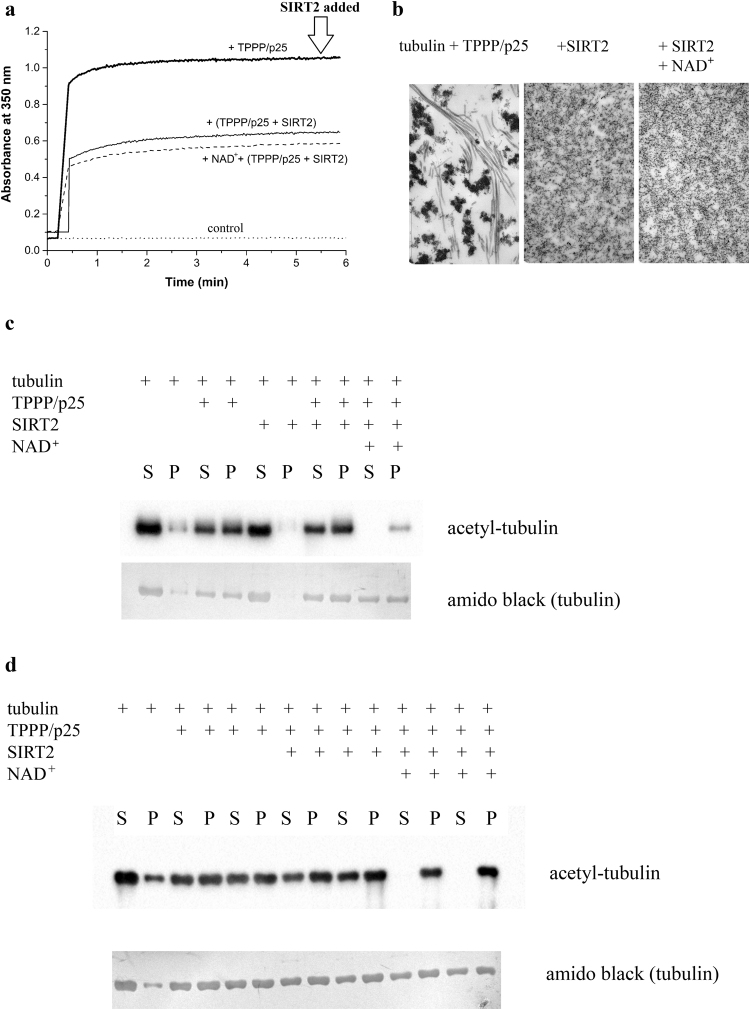
Effect of SIRT2 on the TPPP/p25-induced tubulin polymerization. (**a**) Tubulin polymerization (6 μM) promoted by TPPP/p25 (3 μM) in the absence and presence of inactive or active (with 500 μM NAD^+^) SIRT2 (25 μM). The tubulin polymerization was induced by the addition of TPPP/p25 or TPPP/p25 preincubated with SIRT2. (**b**) Electron microscopic images of the pellet fractions produced by TPPP/p25-induced tubulin assembly in absence and presence of SIRT2 (with NAD^+^) (**a**). (**c**) and (**d**) Western blot images of supernatant (S) and pellet (P) fractions using acetyl-tubulin antibody, when polymerization was induced by TPPP/p25 premixed with SIRT2 (**c**) or SIRT2 was added to the TPPP/p25-assembled tubulin (**d**). All data of the representative experiments can be found in the source data file. The images of the full-length blots are presented in Supplementary Figs S2 and S3.

After revealing the interactions of SIRT2 with TPPP/p25 and/or tubulin, the functional consequences of the heterologous associations were characterized: on one hand, the role of SIRT2 in the TPPP/p25-promoted tubulin assembly; on the other hand, the role of TPPP/p25 in the SIRT2-derived tubulin deacetylation. The following types of experiments were performed as illustrated in Fig. 4: i) TPPP/p25-induced tubulin polymerization as detected by turbidimetry; ii) determination of the partition of proteins in the supernatant and pellet fractions of the samples obtained in the tubulin polymerization assay; iii) quantification of the tubulin acetylation degree by Western blot; iv) visualization of the assembled tubulin structures by electron microscopy.

Previously, we reported that TPPP/p25 promotes tubulin polymerization *in vitro* resulting in intact-like and bundled MTs as well as amorphous aggregates^7^. At cellular level, the ultrastructure of the MT network is highly dependent on the expression level of TPPP/p25: in transiently transfected HeLa cells at low expression level TPPP/p25 is aligned along the MT network without significant morphological alterations; at high expression level both perinuclear cage and aggresome structures are formed^41^.

As shown in Fig. 4, SIRT2 decreases the tubulin polymerization activity of TPPP/p25, when they were premixed, as indicated by the reduced turbidity level; the effect appears to be independent of the presence of NAD^+^ (Fig. 4a). When the same set of experiments was performed, however, SIRT2 was added to the TPPP/p25-assembled tubulin (control curve at 6 min in Fig. 4a), there was no significant decrease in the turbidity (Supplementary Table S2). This finding indicates the resistance of the TPPP/p25-assembled MT ultrastructures against the destructive effect of SIRT2. The ultrastructures formed by the TPPP/p25-promoted tubulin polymerization were visualized by electron microscopy in the presence of either active or inactive SIRT2 (Fig. 4b). The results of this set of experiments unambiguously show that SIRT2, when it was premixed with TPPP/p25, counteracts the TPPP/p25-induced tubulin polymerization independently of its catalytic activity.

The samples were pelleted and analysed by sodium dodecyl sulphate polyacrylamide gel electrophoresis (SDS/PAGE) to visualize the partition of soluble and assembled tubulins complexed with TPPP/p25 and/or SIRT2; in addition, Western blot analysis using acetyl-tubulin antibody was carried out to detect the acetylation levels of the MT ultrastructures formed by the addition of TPPP/p25 premixed with SIRT2 or by addition of SIRT2 to the TPPP/p25-assembled MT. The SDS/PAGE data show that the partition of the proteins in the supernatant and pellet fractions was independent of the presence of NAD^+^, and SIRT2 did not affect significantly the partition of tubulin and TPPP/p25 when it was added to the TPPP/p25-assembled MT (Supplementary Figs S2 and S3). However, the acetylation level was significantly higher in the case of the TPPP/p25-promoted MT ultrastructures (Fig. 4d, Supplementary Fig. S2) as compared to those formed by the addition of TPPP/p25 premixed with SIRT2 (Fig. 4c, Supplementary Fig. S2). The acetylation level of the TPPP/p25-promoted MT ultrastructures was similar to that of the control sample (no SIRT2), these ultrastructures displayed resistance against the deacetylase activity of SIRT2. This finding suggests that certain acetylated sites of the assembled tubulin are not accessible for SIRT2.

### TPPP/p25 inhibits the SIRT2-mediated tubulin deacetylation

**Figure 5:**
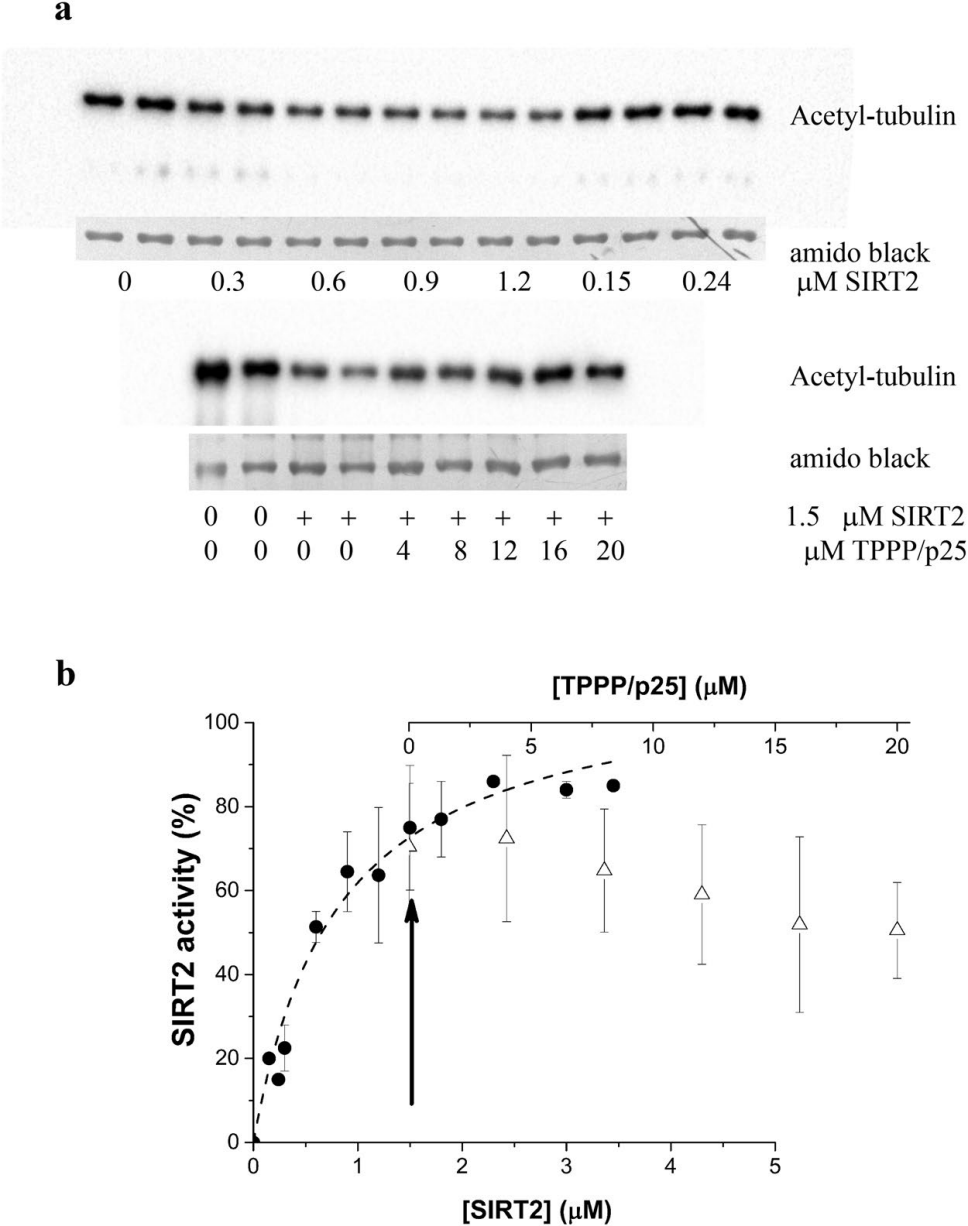
Modest inhibitory potency of TPPP/p25 on the deacetylation of tubulin by SIRT2. (**a**) Concentration-dependent tubulin deacetylation by SIRT2 (●) and the inhibitory effect of TPPP/p25 (∆) on the deacetylase activity of 1.5 μM SIRT2 (indicated by an arrow). The acetylation was determined by Western blot using acetyl-tubulin antibody at 2.64 μM tubulin concentration. (**b**) Quantification of the Western blot data as described in the Materials and Methods. The data are presented as mean ± SD, n = 3 for the SIRT2 concentration serie, and n = 4 for the TPPP/p25 concentration serie at 1.5 μM SIRT2 concentration. Images of the full-length blots/gels are presented in Supplementary Fig. S4.

To study the effect of TPPP/p25 on the SIRT2-derived tubulin deacetylation, the deacetylase activity of SIRT2 was measured on acetyl-tubulin, and quantified by Western blot using specific acetyl-tubulin antibody. Figure 5 and Supplementary Fig. S4 show that SIRT2 decreased the acetylation level of tubulin in a concentration-dependent manner. At a given SIRT2 concentration (1.5 µM), increasing TPPP/p25 concentrations provoked an increase in the tubulin acetylation level suggesting a reduced deacetylation activity of SIRT2 due to its interaction with TPPP/p25. This finding is consistent with the binding (cf. Fig. 2) and the modelling data (cf. Fig. 3), namely, that SIRT2 is involved in the formation of both binary and ternary complexes which display distinct deacetylase activity; in fact, modest inhibitory effect of TPPP/p25 on the SIRT2-mediated tubulin acetylation level can be concluded.

### Mutual effect of TPPP/p25 and chemical inhibitors on the SIRT2-derived deacetylation of tubulin

**Figure 6:**
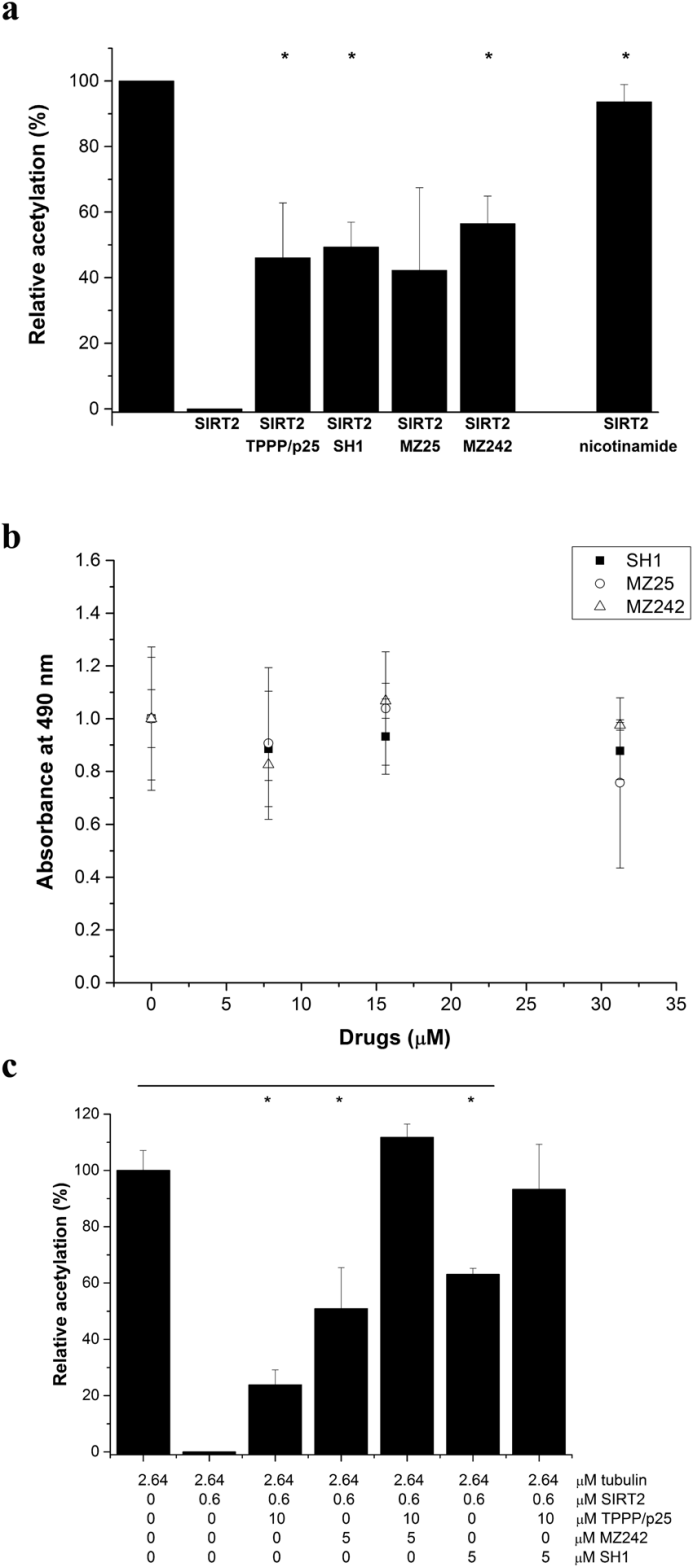
Effect of TPPP/p25 and/or chemical inhibitors on the SIRT2 activity on tubulin. (**a**) Inhibition of deacetylation of 2.64 µM tubulin (black column) by 20 µM TPPP/p25 or chemical compounds; 0.6 µM SIRT2 was added to the acetyl-tubulin. The data are presented as mean ± SD, n = 3–11. *p = 5.56E-06 for TPPP/p25, *p = 1.53E-03 for SH1, *p = 1.38E-03 for MZ242 and *p = 1.60E-03 for 1 mM nicotinamide when the values with and without inhibitor were compared in the case of tubulin as a substrate (two-sided, unpaired Student’s t-test). Images of the full-length blots/gels are presented in Supplementary Fig. S5. (**b**) Effect of the inhibitors on the binding of 125 nM TPPP/p25 to the immobilized SIRT2 detected by ELISA as described in the Materials and Methods. The data are presented as mean ± SD, n = 3. (**c**) Mutual effect of the TPPP/p25 and the chemical inhibitors on the SIRT2 activity (Western blot data with tubulin). The data are presented as mean ± SD. n = 4 for tubulin, tubulin with SIRT2, tubulin with SIRT2 and 10 μM TPPP/p25, tubulin with SIRT2 and 5 μM MZ242, and tubulin with SIRT2 and 10 μM TPPP/p25 and 5 μM MZ242; n = 3 for tubulin with SIRT2 and 5 μM SH1, and tubulin with SIRT2 and 10 μM TPPP/p25 and 5 μM SH1. *p = 1.06E-05 for TPPP/p25, *p = 4.96E-03 for MZ242 and *p = 1.38E-03 for SH1 when the values with and without inhibitor were compared (two-sided, unpaired Student’s t-test). Images of the full-length blots/gels are presented in Supplementary Fig. S6.

To examine whether the small molecule SIRT2 inhibition is affected by the presence of TPPP/p25 as an interacting partner as well as a deacetylase inhibitor, we used a novel class of SIRT2 inhibitors, the SirReals, displaying high inhibitory potency with unique mechanism^36^. In addition, a SirReal-derived PROTAC was used as well, which selectively initiates the proteasomal degradation of SIRT2^39^. Nicotinamide, a well-established inhibitor of SIRT2, was also used as a control^42^. We quantified the effects of the SirReals alone or in combination with TPPP/p25 by Western blot using the endogenous SIRT2 substrate, tubulin, as described in the Materials and Methods. As shown in Fig. 6a (and Supplementary Fig. S5), the efficacy of the different drug-like compounds against the deacetylase activity of SIRT2 is comparable with that of TPPP/p25.

The effect of drug-like inhibitors on the association of SIRT2 to TPPP/p25 was tested by ELISA. Figure 6b shows that the compounds MZ242, MZ25 and SH1 (PROTAC) do not influence the interaction of SIRT2 and TPPP/p25 significantly up to 30 μM, indicating that the interface segment of the heterocomplex is different from the binding site of the chemical inhibitor. Then we evaluated the functional effects of the mutual SIRT2 inhibition by TPPP/p25 and the drug-like inhibitors on the tubulin acetylation level by Western blot using acetyl-tubulin antibody (Fig. 6c, Supplementary Fig. S6). Indeed, a combination of the potential anticancer agents and TPPP/p25 is superior as compared to their individual effects. It should be noted that under *in vitro* conditions in absence of the proteasomal machinery the PROTAC can only function by inhibiting the deacetylase activity of SIRT2 and not as a degrader.

### Localization of the complex of TPPP/p25 and SIRT2 on the microtubule network in HeLa cells

**Figure 7:**
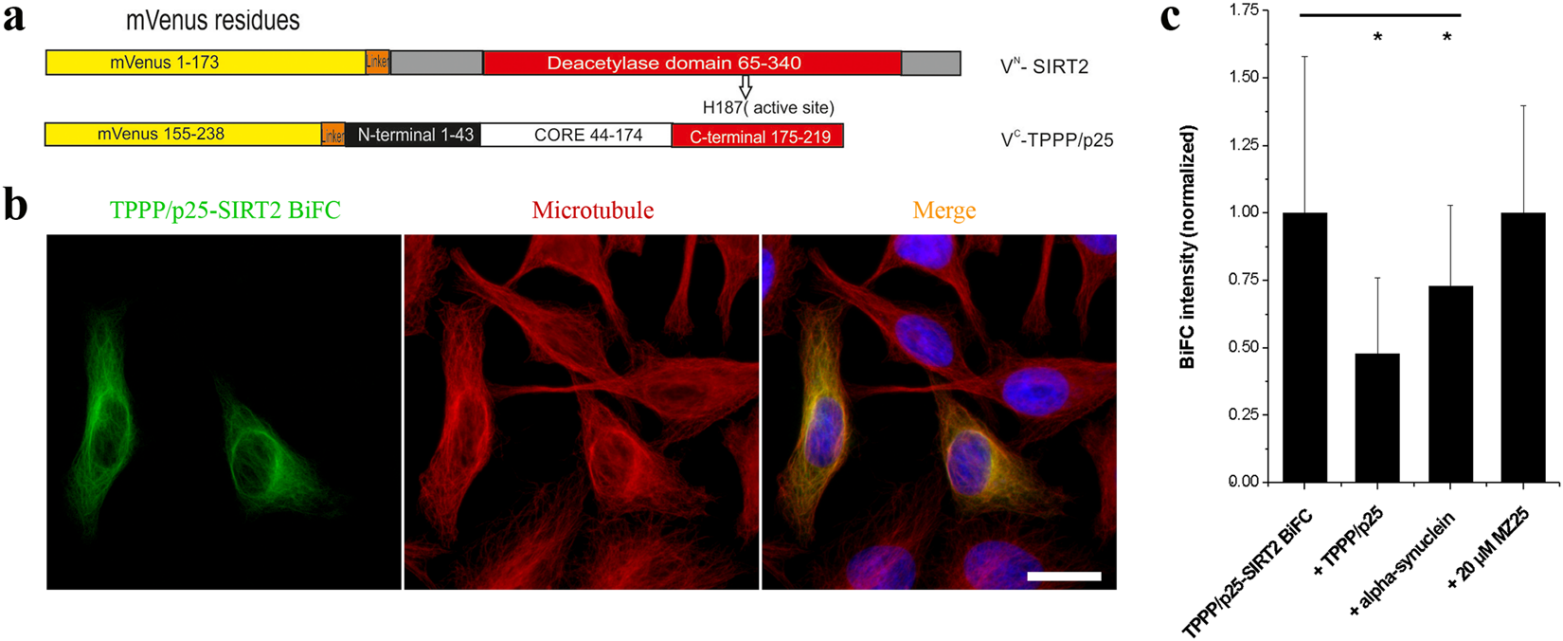
Interaction and localization of TPPP/p25 and SIRT2 in living HeLa cells as detected by immunofluorescence microscopy coupled with BiFC technology. (**a**) Scheme of the applied BiFC constructs. (**b**) Co-localization of the TPPP/p25-SIRT2 complex (green) with the MT network (red). MT network was stained with Alexa546, nuclei was counterstained with DAPI (blue). Scale bar: 10 μm. (**c**) BiFC signal (green) of the assembly of Venus^N^-TPPP/p25 and Venus^C^-SIRT2 and the effect of unlabelled TPPP/p25, α-synuclein and MZ25 was quantified as described in the Materials and Methods. The data are presented as mean ± SD, in each case at least 90 cells were analysed. *p = 2.45E-21 when control (BiFC) cells were compared with those co-transfected with unlabelled TPPP/p25 and *p = 6.76E-06 with unlabelled α-synuclein (two-sided, unpaired Student’s t-test).

Previously we revealed the co-localization of TPPP/p25 with the MT network in transiently transfected HeLa cells resulting in distinct MT ultrastructures^41^. The cancerous HeLa cells express TPPP/p25 endogenously at a very low level; the MT network is virtually deacetylated, however, ectopic expression of TPPP/p25 induces acetylation of the MT network^16^. In order to validate the *in vitro* data obtained with isolated proteins, we performed experiments with HeLa cells using bimolecular fluorescence complementation (BiFC) technology: mVenus vectors of both SIRT2 and TPPP/p25 (V^N^-SIRT2 and V^C^-TPPP/p25) (cf. Fig. 7a) were prepared as described in the Materials and Methods.

As shown in Fig. 7a, the N-terminal and the C-terminal domains of a fluorescent protein (Venus) are fused separately to two partner proteins, SIRT2 and TPPP/p25. The intracellular heteroassociation of the labelled proteins, V^N^-SIRT2 and V^C^-TPPP/p25, brings the two domains of the split fluorescent Venus protein in close proximity, which results in a fluoresce emission upon excitation, a BiFC signal. The control experiment shows that the empty Venus vector does not produce a BiFC signal^43^ (see Supplementary Fig. S7).

Figure 7b shows that the application of BiFC technique coupled with immunofluorescence microscopy enabled not only the visualization of the BiFC complex of SIRT2 and TPPP/p25 (green), but also its localization on the MT network. To further probe the specificity of the interaction of the TPPP/p25-SIRT2 complex and the BiFC signal, the cells were co-transfected with unlabelled TPPP/p25 or α-synuclein. As expected, co-transfection with either unlabelled TPPP/p25 or α-synuclein (interacting partner of TPPP/p25^9,22^) resulted in a significant decrease of the BiFC signal corresponding to the disruption of the Venus complex (Fig. 7c, Supplementary Fig. S7). The established cellular model was also used to validate the effect of the SIRT2 inhibitor, MZ25, on the assembly of SIRT2 and TPPP/p25. In accordance with the *in vitro* data, the addition of MZ25 did not influence the interaction of SIRT2 with TPPP/p25, the BiFC signal intensity was unchanged (Fig. 7c, Supplementary Fig. S7).

**Figure 8:**
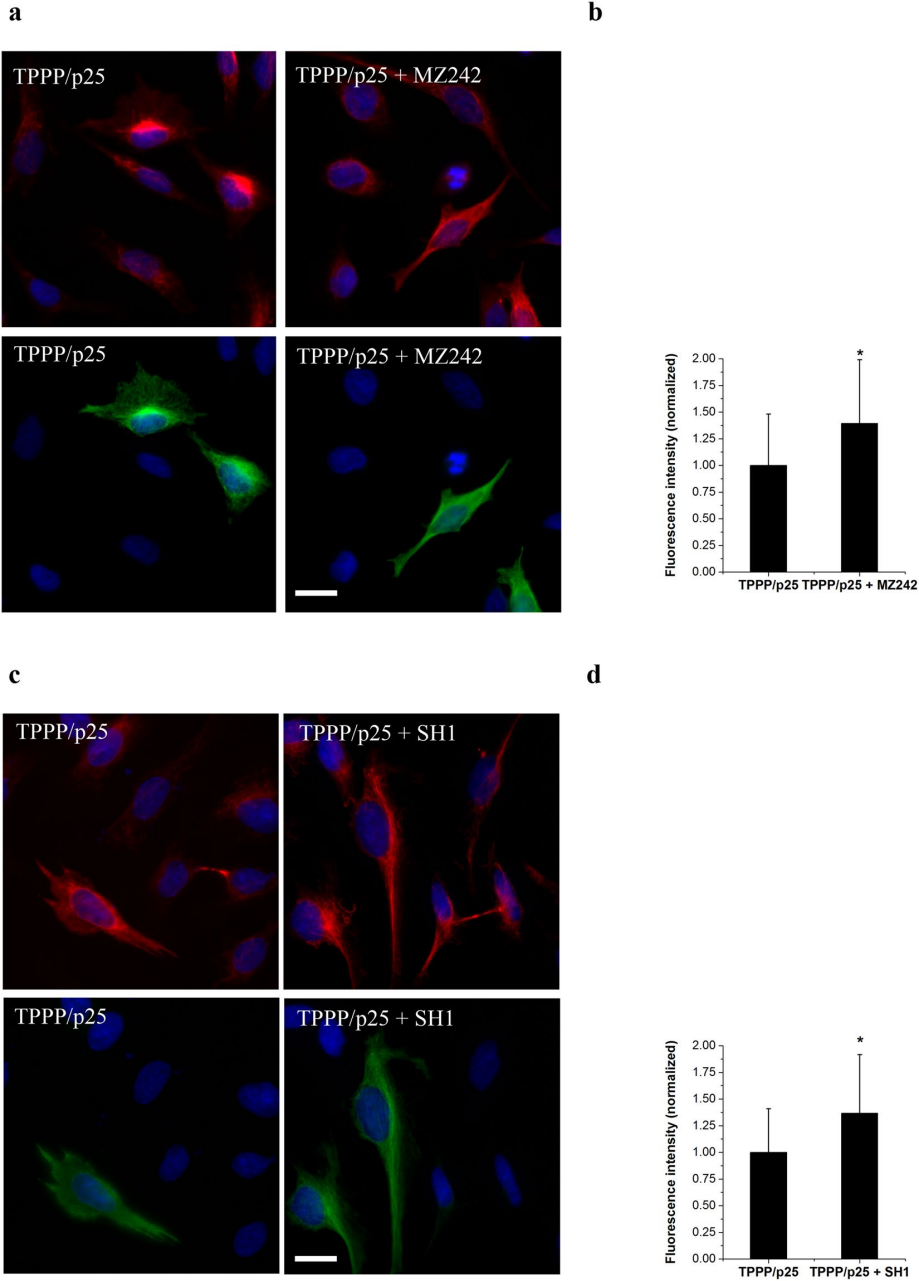
Visualization of the acetylation of the MT network produced by SIRT2 inhibitors (MZ242 and SH1 PROTAC) in TPPP/p25 expressing (green) HeLa cells as compared to the non-transfected cells by widefield immunofluorescence microscopy using specific acetyl-tubulin antibody (red). (**a**) Cells treated with MZ242. (**c**) Cells treated with SH1. Note that the chemical compounds are taken up from the medium, while TPPP/p25 is expressed only in some of the cells. Nuclei are counterstained with DAPI (blue). Scale bar: 5 μm. The acetyl-tubulin signal (red) was quantified in the transfected and non-transfected cells as described in the Materials and Methods. The data are presented as mean ± SD, at least 25 cells (in the case of MZ242, **b**) or 50 cells (in the case of SH1, **d**) were analysed. *p = 9.20E-3 and *p = 3.34E-5 when TPPP/p25 transfected cells were compared with those treated with MZ242 and SH1 as well, respectively (two-sided, unpaired Student’s t-test).

Finally, we have probed the mutual effects of TPPP/p25 and small molecule derived SIRT2 inhibition/degradation (MZ242/SH1), on the acetylation level of the MT network in living cells by immunofluorescence microscopy using acetyl-tubulin antibody (Fig. 8, Supplementary Fig. S8). While the small molecule derived inhibitors/degraders are taken up by all cells from the medium (Supplementary Fig. S8), TPPP/p25 is only expressed in the transiently transfected HeLa cells as identified by immunostaining (green) (Fig. 8a and c). Since the combined acetylation is visible only in the TPPP/p25 expressing cells (few in the images), the immunopositivity for acetyl-tubulin was determined in the transfected cells in the absence and presence of chemical inhibitors on the basis of the fluorescent signal produced by the acetyl-tubulin antibody (red) as described in the Materials and Methods (Fig. 8b and d). According to the quantified data, the tubulin acetylation level is increased with approximately 40% by chemical compounds as compared to the cells transfected with TPPP/p25. Therefore, a competitive effect of TPPP/p25 and small molecule derived degrader SH1 or the inhibitor MZ242 can be ruled out; on the contrary, further hyperacetylation of the MT network due to the combination of TPPP/p25 and chemical inhibitors can be concluded in agreement with the *in vitro* data (cf. Fig. 6c). Recently, it has been revealed that the PROTAC derivative SH1 as a SIRT2 degrader induces process elongation in HeLa cells in addition to the hyperacetylation of the MT network^39^. Now we have revealed that i) this effect is specific for the SH1 degrader since no such effect can be visualized in the case of MZ242 inhibitor (Fig. 8a and c); ii) the SH1 PROTAC effect is maintained in the presence of TPPP/p25; iii) similar morphological alteration has been visualized in the case of tubulin staining using tubulin antibody as for acetyl-tubulin (Fig. 8c, Supplementary Figs S8 and S9) indicating that the MT network is completely reorganized.

## Discussion

Deacetylation of α-tubulin at Lys-40 residue is catalysed by two atypical histone deacetylases, the NAD^+^-dependent SIRT2^25^ and the Zn^2+^-dependent HDAC6^24^, which are potential anti-cancer drug targets^3^; their inhibition provokes the hyperacetylation of the MT network^3^. MAPs such as tau, MAP2c or MAP1B have been suggested to bundle MTs enriched in acetylated α-tubulin leading to resistance against MT depolymerizing agents^44^. Furthermore, the acetylated tubulin residues in these MT bundles are also not accessible to SIRT2^28,44^. TPPP/p25, a recently defined MAP, also induces MT bundling and increases the tubulin acetylation; however, the resistance of the MT network against anti-mitotic agents is caused by the TPPP/p25-mediated cross-linking of the MTs and not by its enhanced acetylation^16^.

Previously we reported that TPPP/p25 interacts with both HDAC6^16^ and SIRT2^29^; however, the functional consequences of the interaction of TPPP/p25 with SIRT2 have not been elucidated. Here we have shown that SIRT2 reduces the TPPP/p25-induced tubulin assembly *in vitro*, it counteracts the tubulin polymerization promoting/MT bundling potency of TPPP/p25 independently of its deacetylase activity (cf. Fig. 4); however, this effect does not occur when SIRT2 is added to the TPPP/p25-assembled tubulin (no turbidity change) (cf. Fig. 4 and Supplementary Figs S2 and S3). Furthermore, the TPPP/p25-promoted ultrastructures, which show resistance against deacetylation, are not accessible for SIRT2 (cf. Fig. 4 and Supplementary Figs S2 and S3).

The assembly of the three partner proteins, tubulin, TPPP/p25 and SIRT2 has been demonstrated i) at molecular level: the binding of tubulin to SIRT2 is stimulated by the presence of TPPP/p25 as detected by ELISA (cf. Fig. 2); ii) at cellular level: the co-localization of the TPPP/p25-SIRT2 complex on the MT network is established by immunofluorescence microscopy coupled with BiFC; iii) by experiment-based mathematical modelling (ternary complex model) (cf. Fig. 3). The knowledge of the specific activity of SIRT2 within the binary and ternary complexes allows one to predict the acetylation level at different concentrations of the proteome components at different conditions. It has been reported that SIRT2 alone does not bind to tubulin^25,45^. This is in agreement with our *in vivo* data (Supplementary Fig. S10). In fact, we were not able to detect intracellular co-localization of SIRT2 with the MT network in HeLa cells. A significant feature of the tubulin acetylation-promoting system has been identified, TPPP/p25, as an interacting partner of both tubulin and SIRT2, can effectively promote the binding of SIRT2 to tubulin likely in a *piggy-back* manner ensuring a fine-tuning mechanism for the regulation of the acetylation level of the MT network. This control could be mediated via conformation changes within the ternary complex leading to the attenuation of SIRT2 deacetylase potency.

Both TPPP/p25^18–20^ and SIRT2^3,31,33,34,46^ are endogenously expressed in the differentiated OLGs, the major constituents of the myelin sheath. The knockdown of TPPP/p25 by siRNA or microRNA impeded the differentiation of OLGs^19^. The knockdown of SIRT2 by siRNA was reported to increase tubulin acetylation and the complexity of cellular arborisation^46^, as well as to block differentiation of OLGs (CG-4 cells)^47^, while its over-expression facilitated cell process growth^47^. However, the abundance of endogenous SIRT2 expression in cultured primary OLG precursors positively correlated with elevated tubulin acetylation and differentiation suggesting its counterbalancing role to prevent uncontrolled acetylation^46^. The detailed mechanism of these complex processes is largely unknown, nevertheless, the regulation of MT stability and dynamics through the interplay of SIRT2 and TPPP/p25 may represent an important mechanism in the cytoskeletal control either during oligodendrogenesis or in mature OLGs. Other MAPs also have been proposed to interact with SIRT2 in a highly specific manner, for example, the protein Furry plays a crucial role in promoting MT acetylation level in the mitotic spindle by inhibiting SIRT2 as demonstrated in HeLa cells^48^.

The control of the acetylation level of the MT network is an important factor for the regulation of MT architecture and maintenance of its integrity^3^, in fact, the non-physiological deacetylation including hyperacetylation has been proposed to play a role in the aetiology of distinct human diseases^3,49,50^. Consequently, the destruction of the cytoskeletal MT system or its over-stabilization leads to cell death. A number of anti-MT drugs have been reported that display such features^51^. The basic problem in cancer therapy is to target specifically the tumour cells, to destroy only their cytoskeletal MT network. Recently, a highly potent and SIRT2 selective inhibitor class, the SirReals, has been discovered, which provokes hyperacetylation of the MT network^36^. Based on these inhibitors, a PROTAC was developed that was shown to induce the proteasomal degradation of SIRT2 chemically^39^. In contrast to the SirReals, the SirReal-derived PROTAC does not only affect the acetylation level of the MT network, but results in process elongation as well. The novel achievements presented in this paper in relation to the control of the MT acetylation level could contribute to our understanding of the multifarious functions of the MT system as well as may provide a clue for innovative strategy in cancer research.

## Materials and Methods

### Inhibitors

The inhibitors were synthesized according to established procedures: MZ25 (SirReal2)^36^ and MZ242 (cpd 10)^38^ are inhibitors of the enzymatic activity of SIRT2. SH1 is a PROTAC, linking a partial structure of MZ242 and thalidomide via a spacer^39^ (Supplementary Fig. S1). Nicotinamide was included in the SIRT2 assay kit (BPS Bioscience, 50087).

### BiFC plasmids

The BiFC plasmids (pBiFC-VN1-173, pBiFC-VC155-238) were a gift of Prof. Péter Várnai (Semmelweis University, Budapest). The V^C^-TPPP/p25 plasmid containing the insert for human full length TPPP/p25 was prepared as described previously^14,43^. Plasmid V^N^-SIRT2 was produced by inserting the SIRT2 coding region in frame^25^ into pBiFC-VN1-173 by XhoI and BamHI restriction enzymes. The sequences of all construct were verified by restriction mapping and sequencing.

### Cloning of SIRT2

Human recombinant hexaHis-tagged SIRT2 containing residues 50–356 was cloned and expressed in *Escherichia coli* (*E. coli*) BL21 (DE3) cells. The constructs of 50-356 SIRT2 were amplified with 5′ primer containing a NdeI site with sequence: 5′- GCACCATATGGACAGCCTGGGCAGCCAGAAGG - 3′ and 3′ primer containing a XhoI site with sequence 5′-GCAATCTCGAGCGACTGGGCATCTATGC - 3′. The resulting PCR fragments were inserted into the NdeI and XhoI sites of the pET21c vector and were confirmed by sequencing.

### Expression and purification of human recombinant wild type TPPP/p25 and SIRT2

The C-terminal hexaHis fusion proteins were expressed in *E. coli* BL21 (DE3). TPPP/p25 was isolated on HIS-Select™ Cartridge (Sigma-Aldrich) as described previously^43^.

SIRT2 was prepared as described by Sun and co-workers^52^. Briefly, the ampicillin-resistant cells were grown in LB containing 100 mg/l ampicillin, 1 mM MgCl_2_ and 5 µM ZnCl_2_ at 37 °C, then harvested by centrifugation (20 min, 4 °C, 2000 *g*). The pellet fraction was resuspended in phosphate buffer (50 mM Na_2_HPO_4_, 300 mM NaCl, pH 8.0 containing 10 μM 4-(2-aminoethyl) benzenesulfonyl fluoride hydrochloride, 1 mM benzamidine, 1 μg/ml pepstatin and 1 μg/ml leupeptin) and lysed by sonication. Following centrifugation (25 min, 4 °C, 39,000 *g*), the supernatant containing the soluble proteins was affinity purified by Ni^2+^ affinity gel. The bound protein was eluted by 50 mM Na_2_HPO_4_, 300 mM NaCl, pH 8.0 containing 50 mM imidazole. Then the protein was concentrated and stored in 50 mM tris(hydroxymethyl)aminomethane, 200 mM NaCl (pH 8.0), 10% glycerol and 1 mM dithioerythritol.

Concentration of TPPP/p25 and SIRT2 was determined at 280 nm by the extinction coefficients evaluated by ProtParam (http://web.expasy.org/protparam/): 10,095 M^−1^*cm^−1^ for TPPP/p25 and 33203 M^− 1^*cm^− 1^ for SIRT2, respectively.

### Tubulin preparation

Tubulin was prepared from bovine brain according to the method of Na and Timasheff^53^. It is important to note that the preparation was carried out without adding zinc cation or NAD^+^ to the buffer compositions used for the preparation of cell extracts or to the ammonium sulphate precipitations.

### CD measurements

CD measurements were performed on a Jasco J-720 spectropolarimeter at 20 nm/min scan rate, 8 s time constant and 1 nm step size in 10 mM phosphate buffer, pH 7.4 at room temperature. The path length was 0.1 cm. Protein concentrations were 4 μM for TPPP/p25, 1 μM for tubulin and 16 μM for SIRT2. The difference spectrum was obtained by subtracting the spectra of the individual proteins from that of the mixture of two proteins. Mean molar ellipticity per residue (MRE) in degrees square centimetre per decimole was calculated according to the following equation: MRE = *Θ*_*m*_/(10*n*c*l), where *Θ*_*m*_ is the measured ellipticity (millidegrees), *n* is the number of amino acid residues in a protein, *c* (mol) is the concentration and *l* (cm) is the path length of the cell. Data from three independent measurements were averaged.

### ELISA

ELISA experiments were performed as described previously^22^. Briefly, the plate was coated with 5 μg·mL^−1^ SIRT2 (or other protein as indicated in the legends of the figures) overnight in phosphate buffered saline (PBS), then the interacting protein at a serial dilution (TPPP/p25 or tubulin) was added. The binding of the protein to the immobilized one was quantified by specific monoclonal TPPP/p25^54^ or tubulin (Sigma T9026) antibodies followed by addition of the secondary anti-mouse IgG conjugated to HRP (Sigma A2554), and the immunocomplex was quantified using o-phenylenediamine with hydrogen peroxide as substrate solution; absorbance was read at 490 nm with an EnSpire Multimode Reader (Perkin Elmer). The binding constants (K_d_) were evaluated from the saturation curves by non-linear curve fitting assuming single binding site hyperbola model using the Origin 8.0 software.

In the case of the competitive ELISA experiments, SIRT2 was immobilized on the plate, tubulin was preincubated with TPPP/p25 at different concentrations, then the binding of TPPP/p25 and that of tubulin were detected by specific TPPP/p25 and tubulin antibodies, respectively. In another set, 125 nM TPPP/p25 was preincubated with MZ25, MZ242 or SH1 at different concentrations, and the binding of TPPP/p25 to the immobilized SIRT2 was detected.

### Turbidimetry and pelleting experiments

The assembly of tubulin (6 μM) was assessed in polymerization buffer (50 mM 2-(N-morpholino)ethanesulfonic acid buffer, pH 6.6, containing 100 mM KCl, 1 mM dithioerythritol, 1 mM MgCl_2_, and 1 mM ethylene glycol tetraacetic acid) at 37 °C in the presence of TPPP/p25 (3 μM) with/or without SIRT2 (25 μM) and NAD^+^ (500 μM). The tubulin polymerization was induced by the addition of TPPP/p25 or TPPP/p25 premixed with SIRT2. When indicated, SIRT2 was added to the TPPP/p25-assembled tubulin at 6 min; after incubation for further 6 min, the absorbance of samples was measured. The optical density was monitored at 350 nm by a Cary 100 spectrophotometer (Varian, Walnut Creek, Australia).

In the pelleting experiments the polymerized samples (100 μl aliquot) were centrifuged (15 min, 37 °C, 17000 *g*), and the pellet (P) and the supernatant (S) fractions were separated. The pellet fractions were resuspended in 100 μl polymerization buffer. The fractions were analysed by SDS/PAGE, Western blot using acetyl-tubulin antibody (Sigma T6793), and electron microscopy. For SDS/PAGE and Western blot experiments, MM indicates the molecular weight marker (PageRuler Prestained Protein Ladder, Thermo Scientific 26616).

### Electron microscopy

For electron microscopy, MT-containing samples were pelleted by centrifugation (15 min, 37 °C, 17000 *g*), and the pellets were fixed in a mixture of 2% glutaraldehyde, 0.2% tannic acid in 0.1 M sodium cacodylate, pH 7.4, for 1 h at room temperature and for 24 h at 4 °C, and then embedded in Durcupan (Fluka, Switzerland). Ultrathin sections were contrasted with Reynold’s lead citrate and examined and photographed in a Jeol JEM-1011 electron microscope, operating at 60 kV, equipped with Olympus Morada CCD camera using Olympus iTEM (TEM imaging platform) software.

### SIRT2 activity assays

#### Tubulin

SIRT2 was incubated with 2.64 μM tubulin, 0.5 mM NAD^+^, 0.1 mg/ml bovine serum albumin without or with TPPP/p25 and/or drugs in assay buffer (BPS Bioscience, 50031) for 1 h at 37 °C. Nicotinamide (1 mM) was used as a positive control of SIRT2 inhibitors^42^. The samples were analysed by 13.5% SDS/PAGE, stained with Coomassie Brilliant Blue R-250 containing 2-mercaptoethanol. The same samples were also electrotransferred onto Immobilon-P^SQ^ transfer membranes and subjected to Western blot. The blot was developed using a monoclonal mouse antibody against acetylated α-tubulin at Lys-40 (1:5000, clone 6-11B-1, Sigma T6793), antibody binding was revealed by anti-mouse IgG-peroxidase conjugate (Fc-specific), (1:5000, Sigma A2554). Peroxidase reaction was detected using Immobilon Western substrate (Millipore) by a Bio-Rad ChemiDoc MP Imaging system and its ImageLab 4.1 software. Then amido black solution (0.1% w/v amido black, 25% v/v isopropanol and 10% v/v acetic acid) was applied to stain the protein bands on the membrane for 1 min. Intensity of bands was analysed by ImageJ 1.49 using Measure command and subtracting background values. Relative acetyl-tubulin level corresponds to the difference measured in absence and the presence of 0.6 μM SIRT2.

### Cell culture and manipulation

HeLa cells (ATCC® CCL-2™, American Type Culture Collection) were grown in Dulbecco’s modified Eagle’s medium supplemented with 10% fetal calf serum and 100 μg/ml kanamycin in a humidified incubator at 37 °C with 5% CO_2_. Cells were grown on 12-mm-diameter glass coverslips for microscopic analysis. For the evaluation of the Venus signal, HeLa cells were transfected with the full length Venus (Addgene) or its fragments V^N^1–173 and V^C^155–238 (empty Venus) or V^C^-TPPP/p25 and V^N^-SIRT2 (0.3 μg of each plasmid), in the case of competition with an additional 0.1 µg TPPP/p25^41^ or α-synuclein construct (OriGene); Turbofect (Invitrogen) transfection reagent was used according to the manufacture’s protocol. The cells were treated with 20 μM MZ25 for 3 hours where indicated. After treatment, the cells were fixed with −20 °C methanol for 10 min, followed by postfixation with 4% formaldehyde (Sigma) for 2 min. After washes with PBS (3 × 10 min), samples were blocked for 30 min in PBS with 0.1% Triton X-100 (Sigma) containing 5% fetal calf serum. Mouse monoclonal tubulin (Sigma T9026), rat polyclonal TPPP/p25^21^, rabbit polyclonal SIRT2 (abcam ab67299), Alexa 546 conjugated mouse, rat and rabbit secondary antibodies (Thermo Fisher Scientific A11030, A11081 and A11010) were used for the detection of the tubulin, TPPP/p25 and SIRT2 signal. Nuclei were counterstained with 4′,6-diamidino-2-phenylindole (DAPI). In another experiment, HeLa cells were transfected with *TPPP/p25* for 24 hours using the same protocol, then inhibitors (10 µM MZ242 or SH1) were added to the samples for the last 3 hours of the experiment. Samples were fixed and processed as above. TPPP/p25-transfected cells were detected with a polyclonal anti-TPPP/p25 serum developed in rat^21^, and labelled by anti-rat Alexa 488 (Thermo Fisher Scientific A11006). Acetyl-tubulin was detected as above. Images of the mounted samples were acquired on a Leica DM IL 500 microscope equipped with Leica DFC 395 FX camera and HBO 100w lamp. Red channel (acetyl-tubulin) or green channel (BiFC) experiments were detected with fixed exposition parameters. The equipment software was Leica Application Suite 4.4.0. Chroma UV filter set (No. C40888), Chroma 41028 HQ NB GFP filter set (No. C21116), Chroma 41028 Y GFP filter set (No. C2117) and Leica filter N2.1 (No. 513832) was used for DAPI, Alexa 488 (and EGFP), BiFC and Alexa 546 signal acquisition, respectively, using a HCX FL Fluotar 40x/0.75 (dry) objective. RGB images were created using Adobe Photoshop CS2 by copying the original greyscale microscopic images (1600 × 1200 pixels, 600 dpi, 8-bit tiff images) into the corresponding red, green, or blue channel, respectively. A minimal background correction was applied by the same way for channels of the selected subpictures. In the presented images, original pixel density was changed to 450 dpi without recalculation.

For the quantitative analysis of the images, the pictures were taken under constant exposure parameters. The determination of the BiFC signal (green, Fig. 7, in each case at least 90 cells were analysed) and the acetyl-tubulin signal (red, Fig. 8, in each case at least 25 cells were analysed) was performed by using the Analyse, Measure option of the National Institutes of Health ImageJ software using the original grayscale pictures without modification. The whole territory of each cell was outlined by the Freehand Line tool and integrated pixel densities were calculated by multiplying the area of each cell with the corresponding average pixel intensity after subtracting the background.

### Mathematical model

All the numerical simulations were performed with Mathematica for Students (version 10.0.1.0) software package (Wolfram Research; http://www.wolfram.com). For the modelling, the experimentally determined dissociation constants of the binary complexes were used. In the case of the ternary complex, it was assumed that the binding of tubulin to the TPPP/p25-SIRT2 complex is weaker than to TPPP/p25 alone but stronger than to SIRT2 alone. The set of differential equations (see Supplementary data) were solved numerically, which allowed the determination of the time-dependent complex formation and the concentrations characteristic for the equilibrium.

### Statistics

The error bars represent the standard deviation (SD). Comparisons were performed using the two-sided, unpaired Student’s t-test and values were considered to be significant if the calculated p value was < 0.05 (*).

### Data availability

All data generated or analysed during this study are included in this published article (and its Supplementary Information files).

## Electronic supplementary material

Supplementary Information

Supplementary dataset
