# Supplementary material for: Modulation Of Microtubule Acetylation By The Interplay Of TPPP/p25, SIRT2 And New Anticancer Agents With Anti-SIRT2 Potency
Source: Sci Rep. 2017 Dec 6;7:17070. doi: 10.1038/s41598-017-17381-3 (PMC5719079; doi:10.1038/s41598-017-17381-3)
Supplement: Supplementary file 1 — Supplementary Information [file 41598_2017_17381_MOESM1_ESM.pdf]

MODULATION OF MICROTUBULE ACETYLATION BY THE INTERPLAY OF TPPP/p25, SIRT2, AND NEW ANTICANCER AGENTS WITH ANTI-SIRT2 POTENCY

[1]Adél Szabó[#], [1]Judit Oláh[#], [1]Sándor Szunyogh, [1]Attila Lehotzky, [1]Tibor Szénási, [1]Marianna Csaplár, [2,3]Matthias Schiedel, [4]Péter Lőw, [2]Manfred Jung, [1]Judit Ovádi*

[1]Institute of Enzymology, Research Centre for Natural Sciences, Hungarian Academy of Sciences, Budapest H-1117, Hungary; [2]Institute of Pharmaceutical Sciences, University of Freiburg, 79104 Freiburg im Breisgau, Germany; [3]Department of Chemistry, Chemistry Research Laboratory, University of Oxford, OX1 3TA Oxford, United Kingdom; [4]Department of Anatomy, Cell and Developmental Biology, Eötvös Loránd University, Budapest H-1117, Hungary

SUPPLEMENTARY MATERIAL

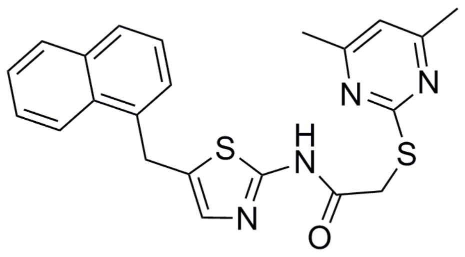

MZ25

IC_{50}(Sirt1): > 100 µM
IC_{50}(Sirt2): 0.44 µM
IC_{50}(Sirt3): > 100 µM

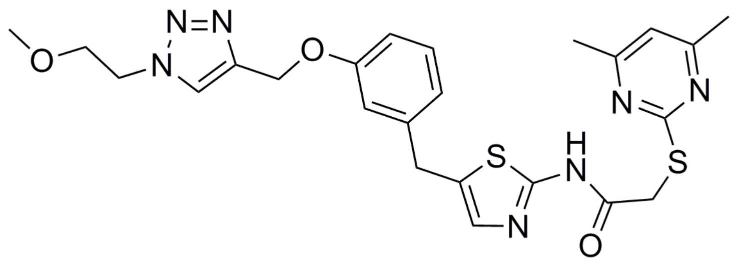

MZ242

IC_{50}(Sirt1): > 100 µM
IC_{50}(Sirt2): 0.12 µM
IC_{50}(Sirt3): > 100 µM

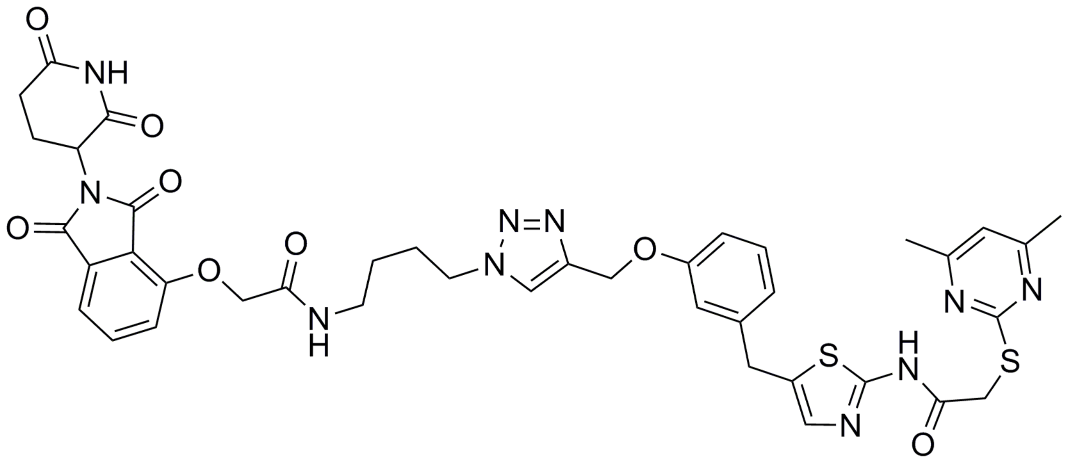

SH1

IC_{50}(Sirt1): > 100 µM
IC_{50}(Sirt2): 0.25 µM
IC_{50}(Sirt3): > 100 µM

Supplementary Fig. S1 - Chemical structures and inhibition data of the SirReal inhibitors (MZ25 and MZ242) and the SirReal-derived PROTAC (SH1).

SDS/PAGE

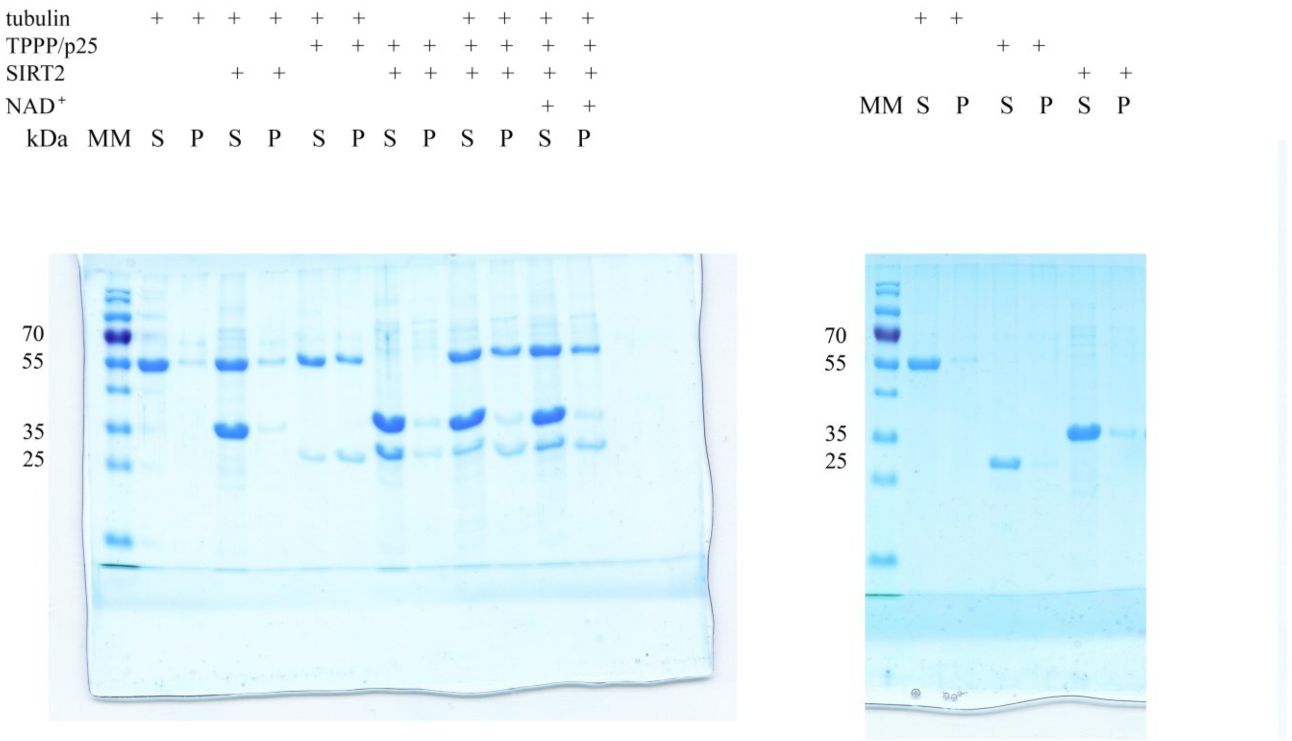

Western blot

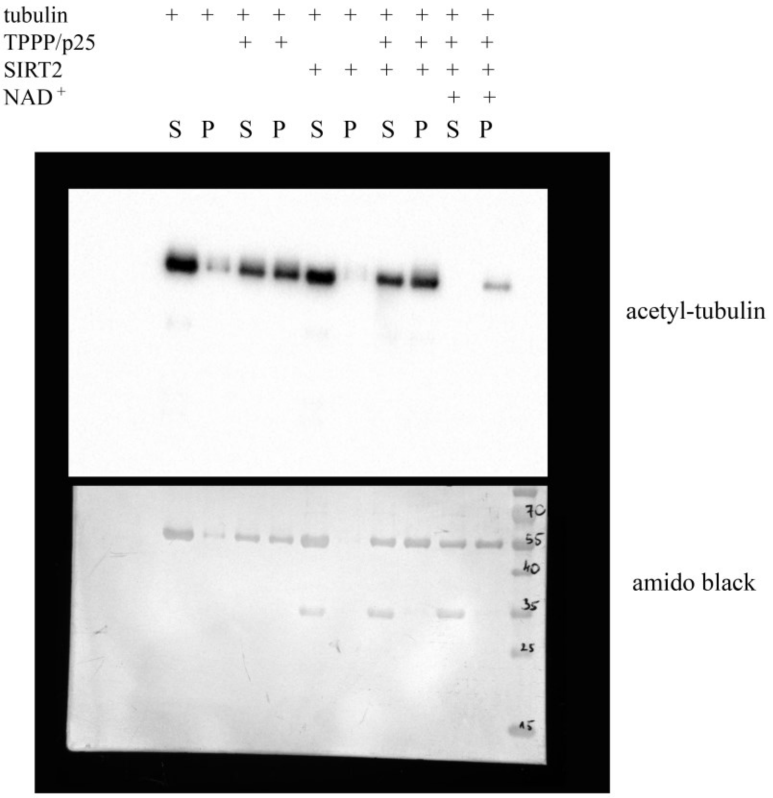

Supplementary Fig. S2 – Full-length SDS/PAGE and Western blot images with molecular weight marker (MM) (Fig. 4c). The tubulin polymerization was induced by the addition of TPPP/p25 or TPPP/p25 preincubated with SIRT2.

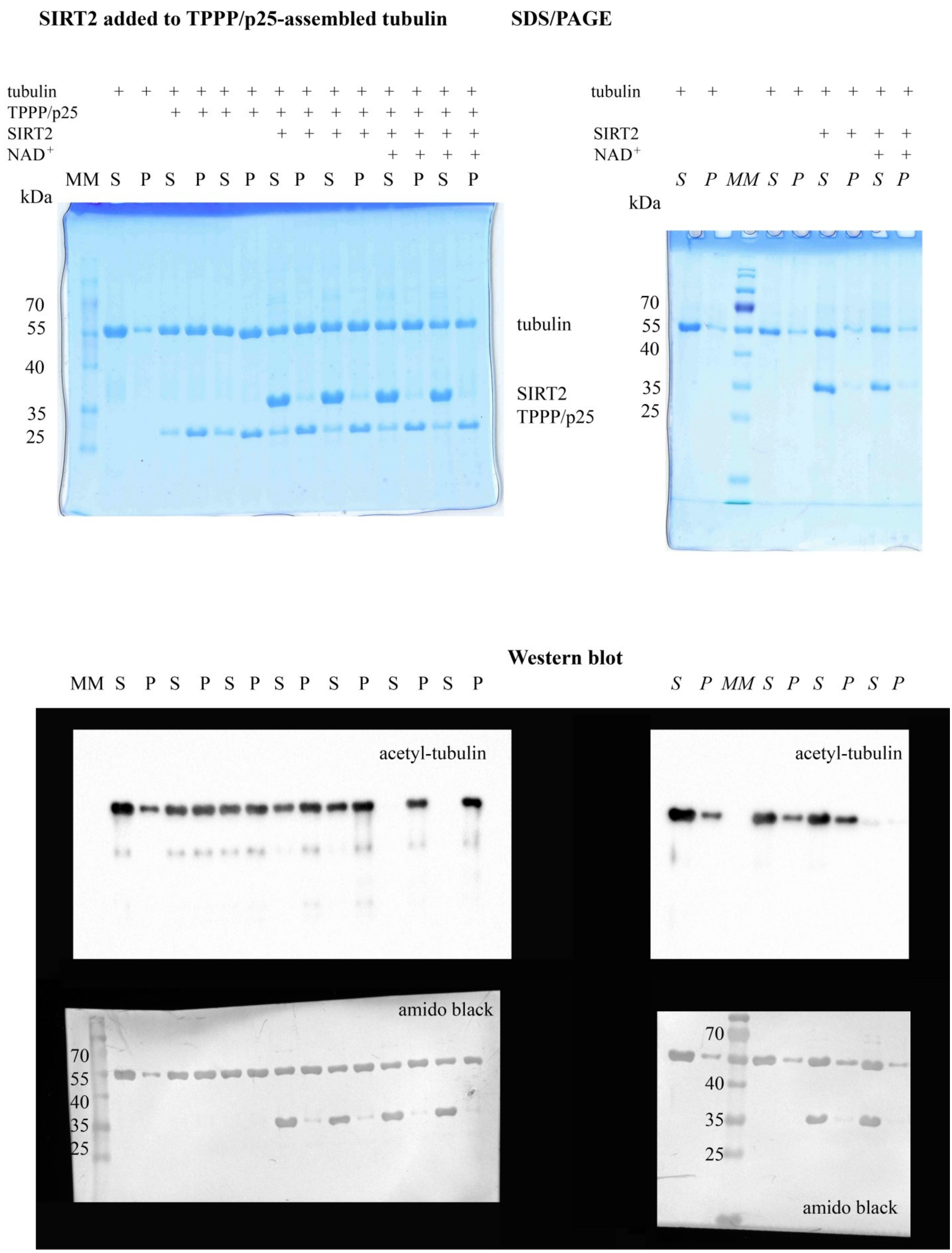

Supplementary Fig. S3 – Effect of SIRT2 on the TPPP/p25-induced tubulin polymerization; SIRT2 was added at constant turbidity (as indicated by an arrow in Fig. 4a). Full-length SDS/PAGE (supernatant (S) and pellet (P) fractions) and Western blot images with molecular weight marker (MM) (Fig. 4d).

SDS/PAGE

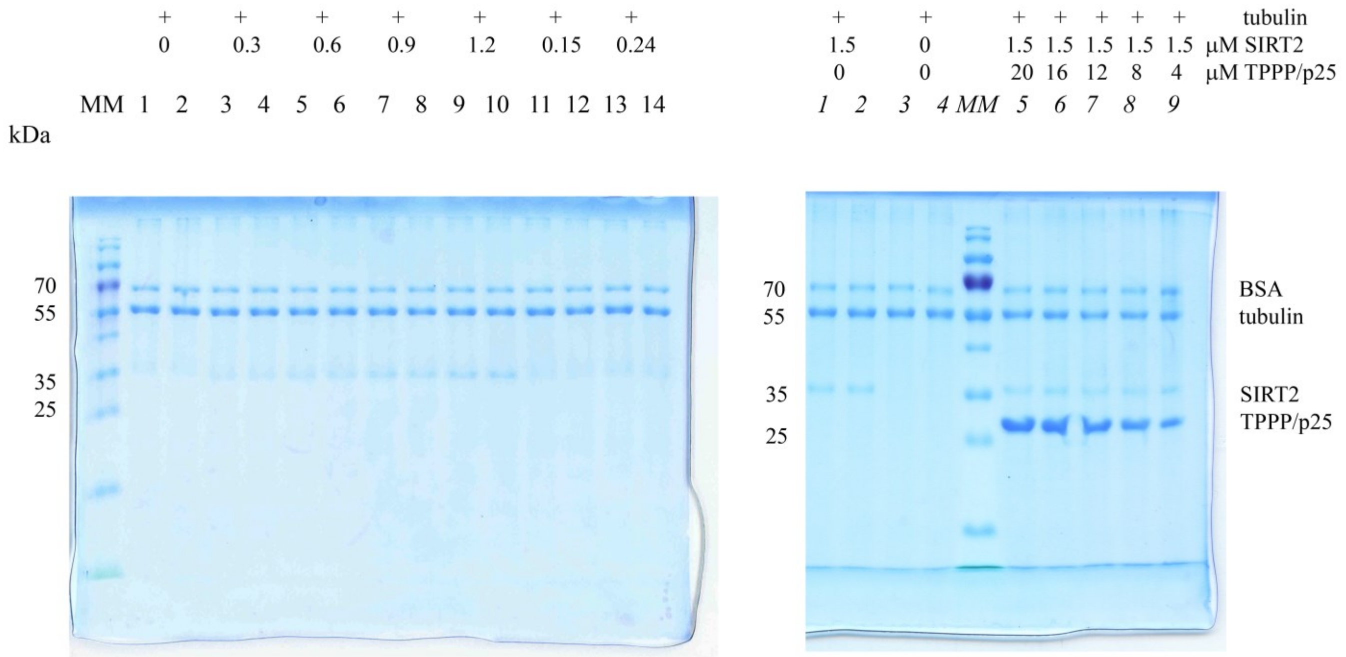

Western blot

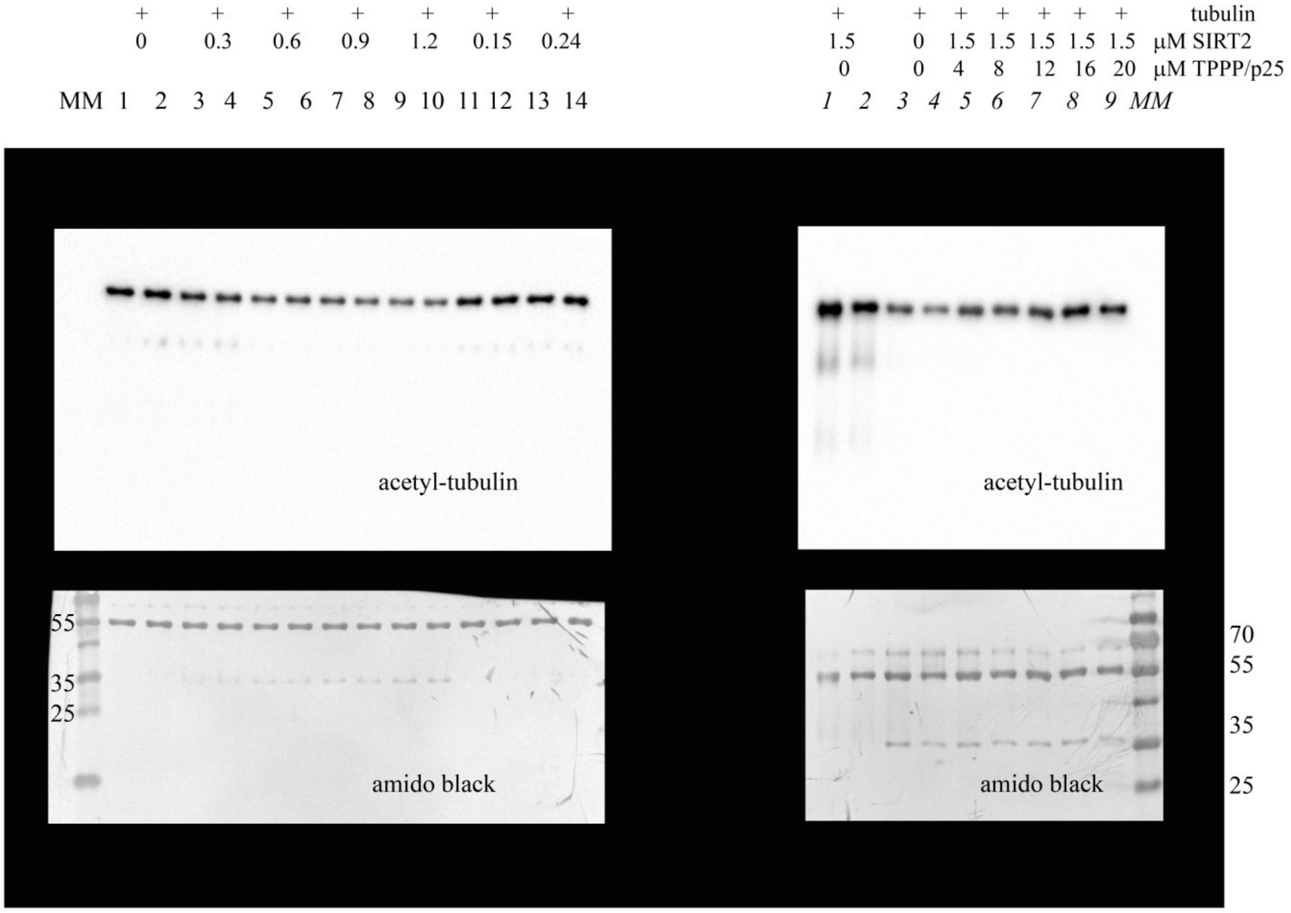

Supplementary Fig. S4 – Modest inhibitory potency of TPPP/p25 on the deacetylation of tubulin by SIRT2. Full-length SDS/PAGE and Western blot images of the samples (Fig. 5) with molecular weight marker (MM).

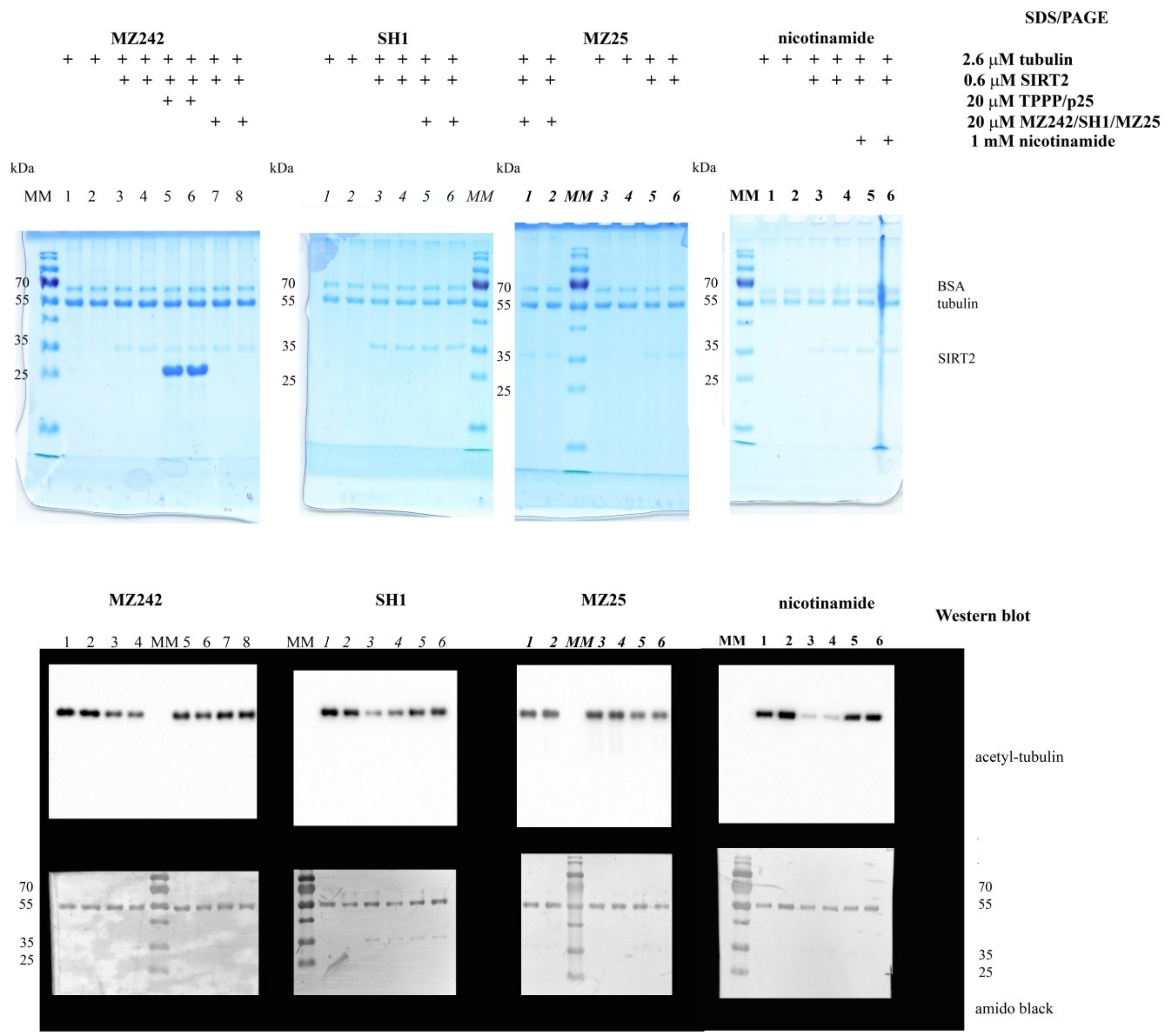

Supplementary Fig. S5 – Effect of TPPP/p25 or chemical inhibitors on the SIRT2 activity. Full-length SDS/PAGE and Western blot images of the samples (Fig. 6a) with molecular weight marker (MM).

MZ242

1-2: tubulin;
3-4: tubulin + SIRT2;
5-6: tubulin + SIRT2 + 10 μM TPPP/p25;
7-8: tubulin + SIRT2 + 5 μM MZ242;
9-10: tubulin + SIRT2 + 10 μM MZ242;
11-12: tubulin + SIRT2 + 5 μM MZ242 + 10 μM TPPP/p25;

SH1

1-2: tubulin;
3-4: tubulin + SIRT2;
5-6: tubulin + SIRT2 + 10 μM TPPP/p25;
7-10: unrelated lanes
11-12: tubulin + SIRT2 + 5 μM SH1;
13-14: tubulin + SIRT2 + 5 μM SH1 + 10 μM TPPP/p25;

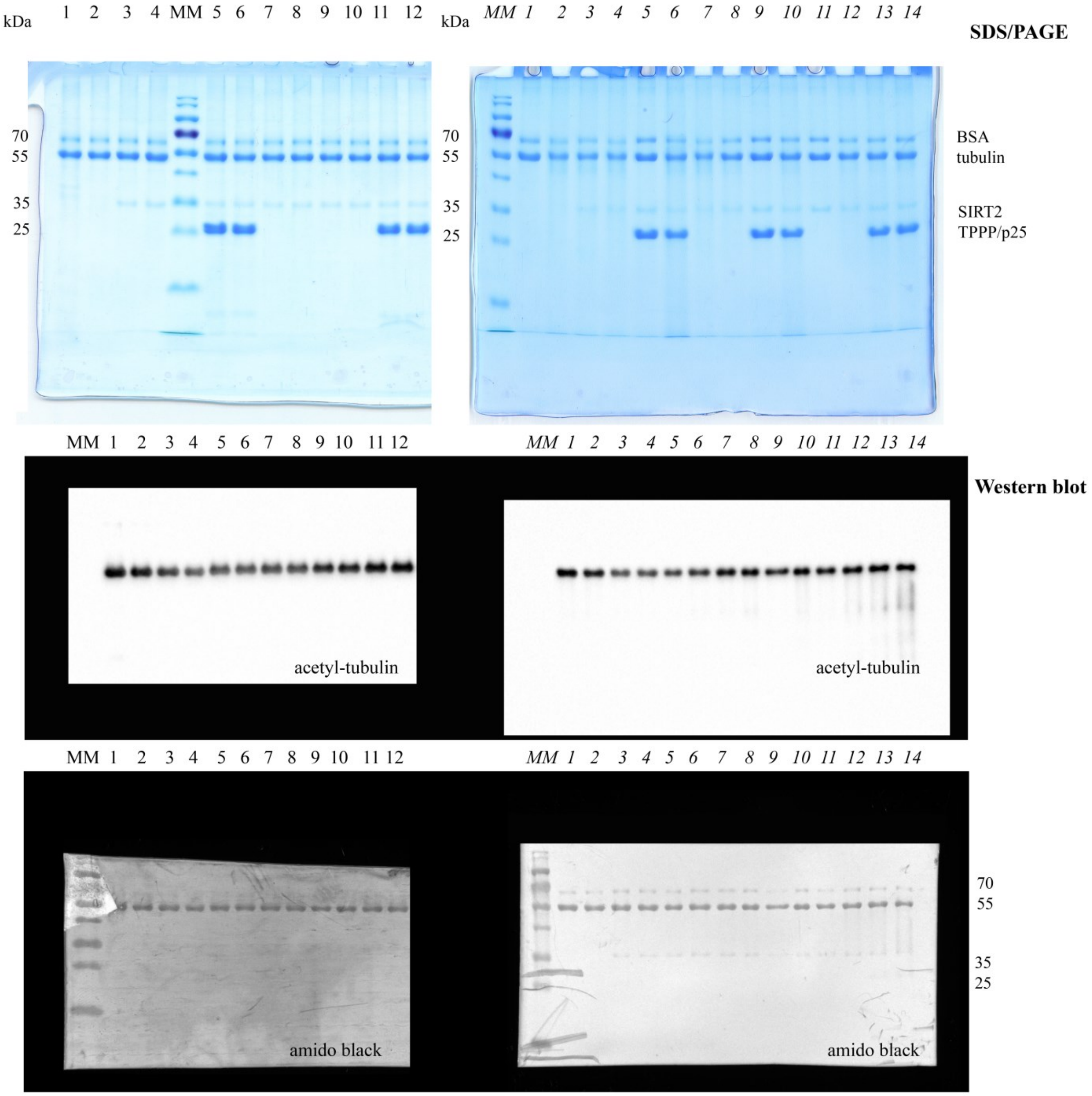

Supplementary Fig. S6 – The mutual effects of TPPP/p25 and chemical inhibitors on the SIRT2 activity. Full-length SDS/PAGE and Western blot images of the samples (Fig. 6c) with molecular weight marker (MM).

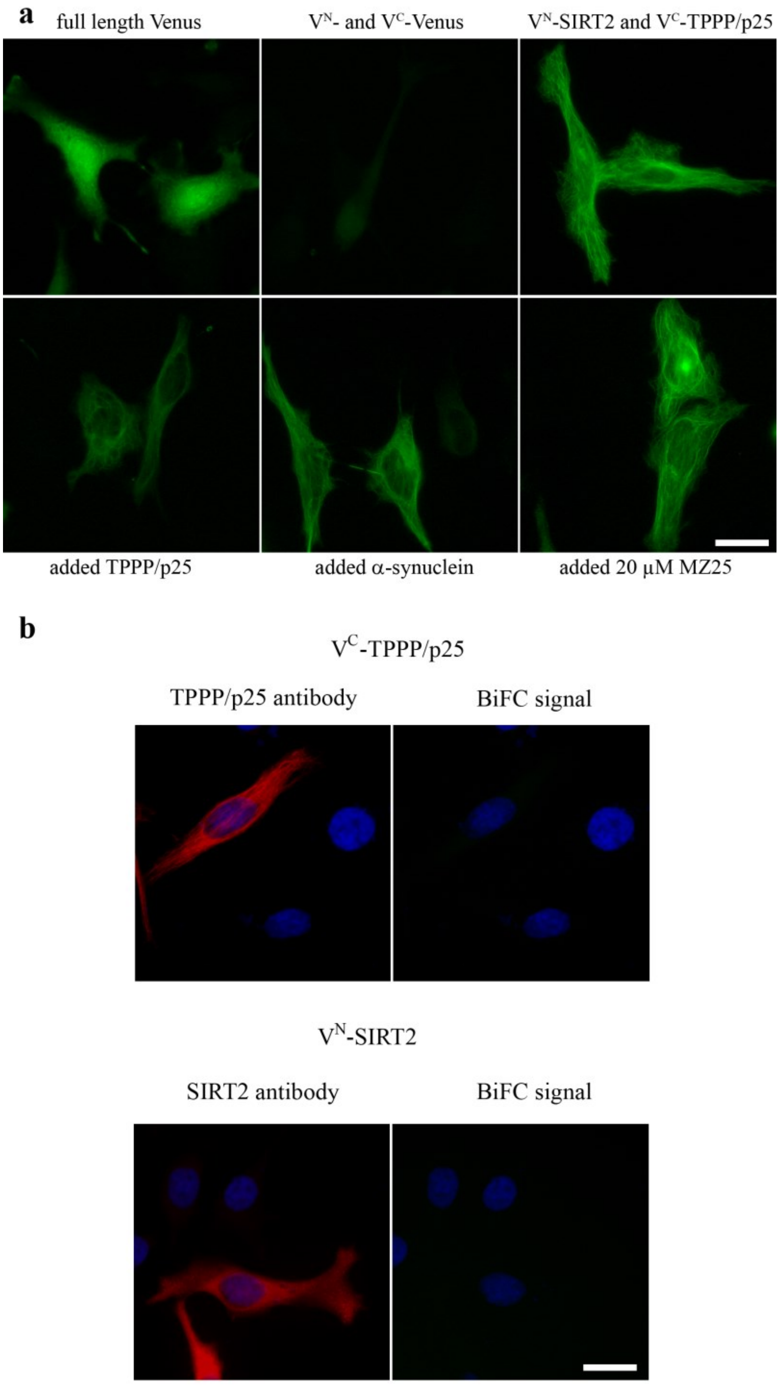

Supplementary Fig. S7 – Representative bimolecular fluorescence signal intensities (green) of the different Venus constructs quantified in Fig. 7c with additional controls. a: Green fluorescent signals of cells transfected with the full length Venus, the two Venus fragments, the two Venus fragments fused with TPPP/p25 and SIRT2 and the competition of the latter with TPPP/p25, α-synuclein or MZ25, respectively. b: BiFC intensities of the Venus fragment fused with V^C-TPPP/p25 or V^N-SIRT2. Note that the V^N and the V^C Venus fragment fused with proteins (on this panel) or not (on panel a) emits negligible fluorescence. The protein expression of the transfected cells was detected by specific antibodies as described in Materials and Methods and visualized with Alexa546 dye. Nuclei was counterstained with DAPI (blue). Scale bar: 10 μm.

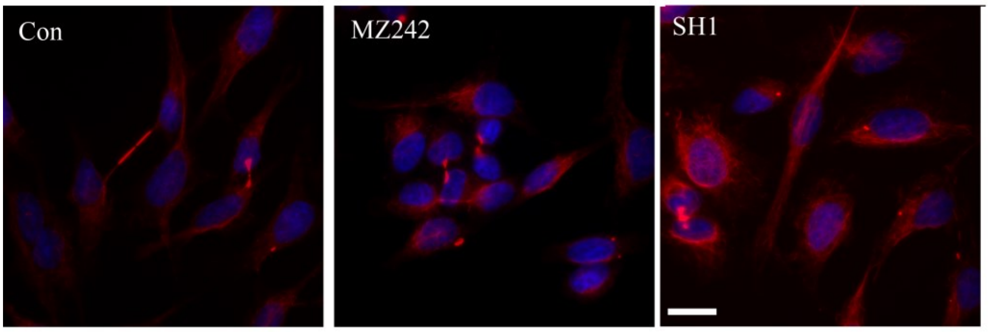

Supplementary Fig. S8 – Visualization of the acetylation of the MT network produced by SIRT2 inhibitors (MZ242 and SH1 PROTAC) in HeLa cells by widefield immunofluorescence microscopy using specific acetyl-tubulin antibody (red). Nuclei was counterstained with DAPI (blue). Scale bar: 5 µm.

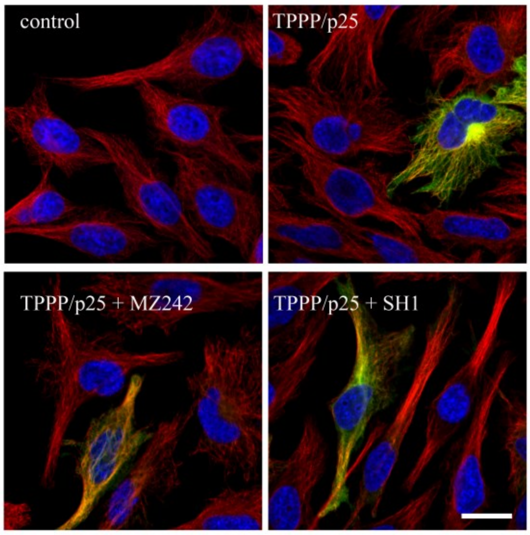

Supplementary Fig. S9 – Visualization of the effects of TPPP/p25 (green) and/or the chemical inhibitors (MZ242 and SH1 PROTAC) on the MT network in HeLa cells by immunofluorescence microscopy using specific tubulin antibody (red). Nuclei was counterstained with DAPI (blue). Scale bar: 10 µm. Pictures was taken a Zeiss 710 confocal microscope with a 40x oiled objective (N.A. = 1.4).

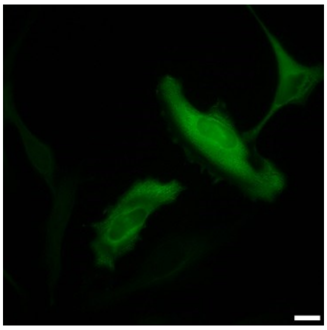

Supplementary Fig. S10 – Localization of EGFP-SIRT2 in HeLa cells as detected by immunofluorescence microscopy. Scale bar: 5 µm.

Mathematical model

Equations and definitions for the mathematical model

Dissociation constants: K_1 = [TPPP/p25][tubulin]/[TPPP/p25-tubulin]

K_2 = [TPPP/p25][SIRT2]/[TPPP/p25-SIRT2]

K_3 = [SIRT2][tubulin]/[SIRT2-tubulin]

K_4 = [TPPP/p25-SIRT2]/[TPPP/p25-SIRT2-tubulin]

Time dependence of the concentration of the different species:

d[TPPP/p25]/dt = – k_{1f}[TPPP/p25][tubulin] + k_{1r} [TPPP/p25-tubulin] – k_{2f}[TPPP/p25][SIRT2] + k_{2r} [TPPP/p25-SIRT2]

d[tubulin]/dt = – k_{1f}[TPPP/p25][tubulin] + k_{1r} [TPPP/p25-tubulin] – k_{3f}[tubulin][SIRT2] + k_{3r} [tubulin-SIRT2] – k_{4f}[tubulin][TPPP/p25-SIRT2] + k_{4r} [TPPP/p25-SIRT2-tubulin]

d[SIRT2]/dt = – k_{2f}[TPPP/p25][SIRT2] + k_{2r} [TPPP/p25-SIRT2] – k_{3f}[tubulin][SIRT2] + k_{3r} [tubulin-SIRT2]

d[TPPP/p25-tubulin]/dt = k_{1f}[TPPP/p25][tubulin] – k_{1r} [TPPP/p25-tubulin]

d[TPPP/p25-SIRT2]/dt = – k_{2f}[TPPP/p25][SIRT2] + k_{2r} [TPPP/p25-SIRT2] – k_{4f}[tubulin][TPPP/p25-SIRT2] + k_{4r} [TPPP/p25-SIRT2-tubulin]

d[tubulin-SIRT2]/dt = k_{3f}[tubulin][SIRT2] – k_{3r} [tubulin-SIRT2]

d[TPPP/p25-SIRT2-tubulin]/dt = k_{4f}[tubulin][TPPP/p25-SIRT2] – k_{4r} [TPPP/p25-SIRT2-tubulin]

Mass conservation equation

$[\text{TPPP/p25}]_{total}$ = [TPPP/p25] + [TPPP/p25-tubulin] + [TPPP/p25-SIRT2] + [TPPP/p25-SIRT2-tubulin]

$[\text{SIRT2}]_{total}$ = [SIRT2] + [tubulin-SIRT2] + [TPPP/p25-SIRT2] + [TPPP/p25-SIRT2-tubulin]

$[\text{tubulin}]_{total}$ = [tubulin] + [TPPP/p25-tubulin] + [tubulin- SIRT2] + [TPPP/p25-SIRT2-tubulin]

Supplementary Table S1. Parameters used for modelling.

Parameter		Value	Unit
total concentration of TPPP/p25	$[\text{TPPP/p25}]_{total}$	0.2	μM
total concentration of tubulin	$[\text{tubulin}]_{total}$	0-10.0	μM
total concentration of SIRT2	$[\text{SIRT2}]_{total}$	0.2	μM
dissociation constant of TPPP/p25 with tubulin#	$K_1 = k_{1r}/ k_{1f}$	0.010	μM
dissociation constant of TPPP/p25 with SIRT2#	$K_2 = k_{2r}/ k_{2f}$	0.030	μM
dissociation constant of tubulin with SIRT2#	$K_3 = k_{3r}/ k_{3f}$	2	μM
dissociation constant of tubulin with TPPP/p25-SIRT2*	$K_4 = k_{4r}/ k_{4f}$	0/0.04	μM

Experimental values are based on the ELISA experiments (see Fig. 2a). *0 μM for the binary and 0.040 μM for the ternary model. The rate constants for the evaluation of the model are derived from the K_d values by taking the k_f values to be 1.

Supplementary Table S2. Effect of SIRT on the TPPP/p25 induced polymerization. Turbidity measurements.

TPPP/p25 preincubated with SIRT2	Absorbance, 350 nm (at 6 min)		
	**mean**	**SD**	**n**
tubulin + TPPP/p25	1.13	0.05	3
tubulin + (TPPP/p25 + SIRT2)	0.78	0.10	3
tubulin + (TPPP/p25 + SIRT2) + NAD^+	0.77	0.13	3
tubulin	0.08	0.01	3
**SIRT2 added to TPPP/p25-assembled tubulin**	**Absorbance, 350 nm (at 12 min)**		
	**mean**	**SD**	**n**
tubulin + TPPP/p25	1.03	0.04	3
(tubulin + TPPP/p25) + SIRT2	1.12	0.03	3
(tubulin + TPPP/p25) + SIRT2 + NAD^+	1.05	0.09	4

The data are presented as mean ± SD.
